# Multidomain Intervention Trial for Preventing Cognitive Decline among Older Adults with Type 2 Diabetes: J-MIND-Diabetes

**DOI:** 10.14283/jpad.2024.117

**Published:** 2024-06-26

**Authors:** T. Sugimoto, A. Araki, H. Fujita, K. Fujita, K. Honda, N. Inagaki, T. Ishida, J. Kato, M. Kishi, Y. Kishino, K. Kobayashi, K. Kouyama, Y. Kuroda, S. Kuwahata, N. Matsumoto, T. Murakami, H. Noma, J. Ogino, M. Ogura, M. Ohishi, H. Shimada, K. Sugimoto, T. Takenaka, Y. Tamura, H. Tokuda, K. Uchida, H. Umegaki, Takashi Sakurai, J-MIND-Diabetes study group

**Affiliations:** 1 Department of Prevention and Care Science, Research Institute, National Center for Geriatrics and Gerontology, Obu, Japan; 2 Department of Medicine, University of Washington, Seattle, Washington USA; 3 Department of Diabetes, Metabolism, and Endocrinology, Tokyo Metropolitan Institute for Geriatrics and Gerontology, Tokyo, Japan; 4 Department of Metabolism and Endocrinology, Akita University Graduate School of Medicine, Akita, Japan; 5 Department of Medical Nutrition, Kagawa Nutrition University, Sakado, Japan; 6 Department of Diabetes, Endocrinology and Nutrition, Graduate School of Medicine, Kyoto University, Kyoto, Japan; 7 Department of General Medicine, Tokyo Medical and Dental University, Tokyo, Japan; 8 Department of Internal Medicine, Hyogo Prefectural Rehabilitation Center at Nishi-Harima, Tatsuno, Japan; 9 Department of Internal Medicine, Nishiwaki Municipal Hospital, Nishiwaki, Japan; 10 Department of Diabetes and Metabolic Diseases, Asahi General Hospital, Asahi, Japan; 11 Department of Diabetes Medicine, Hyogo-Chuo National Hospital, Sanda, Japan; 12 Department of Internal and Cardiovascular Medicine, Tarumizu Chuo Hospital, Tarumizu Municipal Medical Center, Tarumizu, Japan; 13 Department of Data Science, Institute of Statistical Mathematics, Tokyo, Japan; 14 Department of Cardiovascular Medicine and Hypertension, Graduate School of Medical and Dental Sciences, Kagoshima University, Kagoshima, Japan; 15 Center for Gerontology and Social Science, Research Institute, National Center for Geriatrics and Gerontology, Obu, Japan; 16 Diabetes Center, Ohta Nishinouchi Hospital, Koriyama, Japan; 17 Department of Endocrinology and Metabolism, Hospital, National Center for Geriatrics and Gerontology, Obu, Japan; 18 Department of Community Healthcare and Geriatrics, Nagoya University Graduate School of Medicine, Nagoya, Japan; 19 Research Institute, National Center for Geriatrics and Gerontology, Obu, Japan; 20 Department of Cognition and Behavior Science, Nagoya University Graduate School of Medicine, Nagoya, Japan; 21 7-430 Morioka, Obu, Aichi, 474-8511 Japan

**Keywords:** Multidomain intervention, cognitive decline, type 2 diabetes, exercise, diet

## Abstract

**Background:**

No multidomain intervention trials have been designed for the prevention of cognitive decline in older adults with type 2 diabetes.

**Objectives:**

To investigate the efficacy of a multidomain intervention in preventing cognitive decline in older adults with type 2 diabetes and cognitive impairment.

**Design:**

Eighteen-month, multi-centered, randomized controlled trial.

**Setting:**

Twelve hospitals in Japan.

**Participants:**

Outpatients with type 2 diabetes aged 70–85 years with cognitive impairment.

**Intervention:**

The multidomain intervention program includes management of metabolic and vascular risk factors, exercise, nutritional counseling, and promotion of social participation. Participants in the control group received usual care and treatment for type 2 diabetes.

**Measurements:**

The primary outcome was the change in a composite score combining several neuropsychological tests from baseline to the 18-month follow-up. To assess the differences in cognitive changes between the intervention and control groups, a mixed-effects model for repeated measures was used.

**Results:**

Between March 13, 2019, and May 8, 2020, 361 participants were screened, and 154 were randomly assigned to either the intervention group (n = 81) or the control group (n = 73). Finally, 110 participants completed the trial. The between-group difference in the composite score changes was 0.068 (95% confidence interval, −0.091 to 0.226). Analyses for secondary outcomes indicated a positive impact of the intervention on memory and indicated that the intervention led to changes in dietary habits with increased intakes of niacin and meat, along with weight reduction compared to the control group.

**Conclusion:**

The multidomain intervention did not demonstrate efficacy in preventing cognitive decline. However, this trial provided proof-of-concept evidence that multidomain interventions may offer cognitive benefits and contribute to changes in dietary behavior and weight reduction in older adults with type 2 diabetes and cognitive impairment. These findings should be confirmed in future studies.

**Electronic Supplementary Material:**

Supplementary material is available in the online version of this article at 10.14283/jpad.2024.117.

## Introduction

**T**ype 2 diabetes has become increasingly prevalent among older people worldwide (1). Numerous studies have demonstrated that diabetes is associated with a higher risk of cognitive impairment and dementia (2). These conditions significantly impact person’s quality of life and overall diabetes management due to poor adherence to complex dietary recommendations, physical exercise, and medications. Consequently, there is an urgent need to develop effective strategies for preventing and managing cognitive decline in older adults with diabetes.

The underlying mechanism of cognitive decline in type 2 diabetes is still unclear, but it is likely that multiple factors are involved. To date, a wide range of potentially modifiable risk factors in type 2 diabetes have been reported in epidemiological studies, including hyperglycemia (3), severe hypoglycemic events (4), glycemic variability (5), physical inactivity (6), nutritional status (weight loss and gain) (7), diet (8), and social participation (9). In this context, several large randomized controlled trials, including the Finnish Geriatric Intervention Study to Prevent Cognitive Impairment and Disability (FINGER) study (10), have investigated the efficacy of multidomain intervention targeting multiple modifiable risk factors simultaneously in preventing cognitive decline in the general older population. However, the results have been varied (11, 12). In people with type 2 diabetes, exploratory analyses from the Look AHEAD study showed that multidomain lifestyle interventions focused on weight loss and increased physical activity have no significant effect on cognition (13), although beneficial effect on white matter hyperintensities, ventricle volumes, and cerebral blood flow was observed (14, 15). Nevertheless, there is still a lack of evidence from multidomain intervention trials specifically designed to prevent cognitive decline in older adults with type 2 diabetes.

The Japan-Multidomain Intervention Trial for Prevention of Dementia in Older Adults with Diabetes (J-MIND-Diabetes) was conducted to investigate the efficacy of the multidomain intervention (consisting of management of vascular risk factors, exercise, nutritional counseling, and promoting social participation) in preventing cognitive decline among older adults with type 2 diabetes. Notably, this trial focused on older adults classified into Category II (mild cognitive impairment [MCI] to mild dementia), who are at high risk of cognitive decline and disabilities, according to the “Glycemic Targets for Elderly Patients with Diabetes” guidelines set by the Japan Diabetes Society/Japan Geriatrics Society (JDS/JGS) Joint Committee (16). The guidelines have significantly impacted clinical practice and have been widely adopted (17). From the perspective of clinical prevention of further cognitive decline, this study can offer initial insights into the efficacy and social implementation of multidomain intervention among individuals with type 2 diabetes.

## Methods

### Study design

The J-MIND-Diabetes is an 18-month, randomized, controlled, multicenter trial conducted at 12 independent hospitals in Japan. The study procedures were reviewed and approved by the Institutional Review Boards at all participating institutions. The study protocol has been previously published (18). This trial was registered in the University Hospital Medical Information Network Clinical Trials Registry (Trial number UMIN000035911) (https://center6.umin.ac.jp/cgi-open-bin/ctr_e/ctr_view.cgi?recptno=R000040908). This trial followed the Consolidated Standards of Reporting Trials guideline (19).

### Participants

Outpatients with type 2 diabetes aged 70–85 years were recruited. They were classified into Category II based on the “Glycemic Targets for Elderly Patients with Diabetes” (16). Accordingly, participants had no or mild impairment of basic activities of daily living but were mildly cognitively impaired, as determined by a score of less than 26 on the Japanese version of the Montreal Cognitive Assessment (MoCA-J) (20) and a score of 21–30 on the Mini-Mental State Examination (MMSE) (18, 21). Those who needed to preclude and/or restrict physical exercise were excluded from this trial. All inclusion and exclusion criteria are presented in the Supplemental Methods (Pages 4–5). The purpose, nature, and potential risks of this trial were fully explained to the participants, and all participants provided written informed consent before participating in the trial.

### Interventions

Participants in the control group received usual care and treatment for type 2 diabetes. In Japan, a joint committee between the JDS and JGS was established in April 2015 to enhance diabetes management in older adults. Subsequently, guidelines specific to older patients with diabetes were developed (16). Additionally, at baseline, they were given general instructions on dementia risk reduction, including information on the benefits of physical activities, maintaining a healthy diet, engaging in cognitive activities, and social participation by using brochures.

The intervention group received multidomain intervention programs, which included the management of metabolic and vascular risk factors, group-based physical exercise, nutritional counseling, and promotion of social participation. To manage metabolic and vascular risk factors, diabetes, hypertension, and dyslipidemia were treated in accordance with the relevant clinical guideline recommendations. Specifically, for management of diabetes (16), participants classified into Category II had an upper limit of the glycemic target (glycated hemoglobin A1c [HbA1c]) of 7.0% for participants not receiving drugs associated with a high risk of severe hypoglycemia. However, for those receiving these drugs, the upper and lower limits of the glycemic target were set at 8.0% and 7.0%, respectively (Supplemental Methods, Page 5).

Participants were encouraged to engage in group-based physical exercise sessions at least once every two weeks, totaling 39 sessions. These sessions were conducted by study physiotherapists or trained instructors and included muscle strength training and postural balance training, aerobic exercise, and dual-task training (18). Participants were also advised to engage in home-based exercise at least twice a week, as well as to self-monitor these activities using study-provided booklets and pedometers. Due to restrictions on group-based physical exercise sessions in all institutes during the coronavirus disease 2019 (COVID-19) pandemic, the participants were given documents and a digital versatile disc to promote home-based exercise at least twice a week. Furthermore, to sustain participant motivation, our study staff recorded pedometer readings, provided feedback during outpatient visits, and conducted follow-up with telephone calls.

Nutritional counseling sessions were individually provided by registered dieticians during each medical examination, approximately once every two months (18). The counseling included guidance on the following aspects: 1) appropriate energy intake; 2) sufficient protein intake to improve physical condition; 3) sufficient intake of green-yellow vegetables and seaweed to ensure a sufficient intake of B vitamins; 4) lifestyle and dietary behavior improvement through smoking cessation and reduced alcohol consumption; 5) a well-balanced diet; and 6) chewing and swallowing function and oral care. Participants were instructed to monitor their body weight, meal start times, and dietary diversity on a daily basis using a study-provided place mat illustrating dietary variety and a dietary diary (18). During each counseling session, the dieticians provided two kinds of foods, which include rice with mixed grains to increase fiber intake, canned mackerel, salmon, and sardine to increase the intake of proteins, eicosapentaenoic acid, and docosahexaenoic acid, fish sausage to increase protein and calcium intake, and cooking recipes.

To promote social participation, participants were requested to go out at least three times a week. Additionally, they were asked to monitor their social activities, including taking care of their grandchildren, engaging in work or volunteer activities, having face-to-face conversations, going shopping or eating out, and participating in organizations or groups (18).

### Outcomes

All cognitive outcomes were assessed trained psychologists (22). The primary outcome of this trial was to measure the change in a composite cognitive score at 18 months compared to baseline. This score was determined by administering several neuropsychological tests, including the MoCA-J (20), MMSE (21), Rey–Osterrieth Complex Figure Test (ROCFT) (23), delayed recall test of a 10-word list derived from the Alzheimer’s Disease Assessment Scale-cognitive subscale (24), Wechsler Adult Intelligence Scale Digit Span (25), Trail Making Test (26), Digit Symbol Substitution Test (25), and the letter word fluency test (26). To calculate the composite score, the Z scores of each neuropsychological test standardized with baseline means and standard deviations for each test from the full analysis set (FAS) were averaged. In the calculation of the composite score, the inverse of the Z scores for the delayed recall test of a 10-word list and the Trail Making Test were used because a lower score on these tests indicates better cognitive function.

The secondary outcomes related to cognitive changes included the change in composite cognitive score from baseline to six months and the change in scores of each neuropsychological test at both the six- and 18-month follow-ups, compared to baseline. Additionally, other secondary outcomes included changes in diabetes-related outcomes (metabolic control determined by HbA1c and continuous glucose monitoring [CGM]-derived metrics, self-reported hypoglycemic events, microangiopathy, and macroangiopathy) and comprehensive geriatric assessment results, including activities of daily living, depressive symptoms, anthropometric measurements, physical performance (gait speed, hand grip strength, and one-leg standing test), physical frailty and sarcopenia, fall risk, nutritional status, social network, social participation, and the number of medications. Furthermore, the intake of nutrients and food groups was assessed by registered dieticians using the Food Frequency Questionnaire based on food groups at the baseline and 18-month follow-up (27). Detailed information about all secondary outcome measurements is presented in the Supplemental Methods (Pages 5–7).

Throughout this trial, serious adverse events (SAE) were monitored for the intervention group, while they were assessed at both the six- and 18-month follow-up times for the control group.

### Sample size

The sample size for this study was calculated based on clinical data from MMSE assessments over 1-year in 83 older adults with both MCI and diabetes classified into Category II at the Memory Clinic of the National Center for Geriatrics and Gerontology independent of the J-MIND-Diabetes. The mean change in the MMSE score among the 83 older adults was found to be −1.45 ± 3.55. Among them, those with glucose levels within the target range (n = 30) showed a mean change in MMSE score of −0.33 ± 2.58. Based on these data, it was hypothesized that the present study would be able to detect differences in the change of cognitive function between the intervention and control groups (−0.33 ± 2.58 vs. −1.45 ± 3.55). Using a two-sided significance level of 5% and a statistical power of 80%, the calculated total sample size required for the study was 242. However, considering an estimated dropout rate of 15%–20% at each institution by the 18-month follow-up, the total required sample size was estimated to be 300 participants (18).

### Randomization and masking

Participants were centrally randomized at a 1:1 allocation ratio into intervention and control groups using stratified permuted-block randomization methods. The following stratification variables were used: 1) institutions (1–12 institutions); 2) age at enrollment (70–77 years vs. 78–85 years); 3) glycemic control relative to the glycemic targets recommended by the JDS/JGS joint committee (within target range vs. above upper limit or below lower limit); and 4) use of drugs potentially associated with severe hypoglycemia such as insulin, sulfonylureas, or glinides (those receiving these drugs vs. those not receiving these drugs). The randomization sequences were generated by a trial statistician (HN) using the Proc Plan function in SAS Version 9.4 (SAS Institute, Cary, NC, USA). Participants and research staff, including investigators and clinicians, at each institution were unaware of the randomization sequences and block size. The research staff members assessing primary outcome data were blinded to the group assignment and were not involved in the intervention programs. However, the research staff members providing intervention programs and the participants themselves were not blinded to their allocation.

### Statistical methods

In this trial, two analysis sets were defined: the FAS and the per protocol set (PPS). The FAS included participants who participated in at least one physical exercise session or received general health instruction, whereas the PPS included participants who were compliant with the study protocol and completed the 18-month follow-up.

The primary efficacy analysis was conducted using the FAS. To assess the differences in cognitive changes at the six- and 18-month follow-up compared to baseline between the intervention and control groups, a mixed-effects model for repeated measures (MMRM) (28) with an unstructured covariance structure was used. This model included groups, time of the visit, group-by-time interaction, and baseline composite cognitive score as explanatory variables. For the secondary continuous outcomes, excluding cognitive function, the Wilcoxon rank sum tests were used to compare the changes in outcomes between the intervention and control groups. For the secondary categorical outcomes, chi-squared tests were used. The frequencies of SAE were compared between the intervention and control groups using the chi-squared test.

Four prespecified subgroup analyses were conducted based on the following factors: age at enrollment (70–77 years vs. 78–85 years), baseline glycemic control (within target range vs. above upper limit or below lower limit), use of drugs potentially associated with severe hypoglycemia (those receiving these drugs vs. those not receiving these drugs), and apolipoprotein E (APOE) phenotype (APOE ε4 carrier vs. APOE ε4 noncarrier). Additionally, another prespecified subanalysis was conducted based on the adherence rate to group-based physical exercise sessions, which included three groups: an adherence rate of 50% or greater, an adherence rate less than 50%, and a control group.

A post-hoc subgroup analysis based on sex was conducted using MMRM with the same covariates mentioned previously. Additionally, a subgroup analysis was conducted, excluding participants with an MMSE score of <24, which is suggestive of mild dementia.

All statistical analyses were conducted by a study statistician (HN) using SAS 9.4 (SAS Institute, Cary, NC, USA). P-values of less than 0.05 were considered statistically significant.

## Results

### Participant characteristics

**Figure 1:**
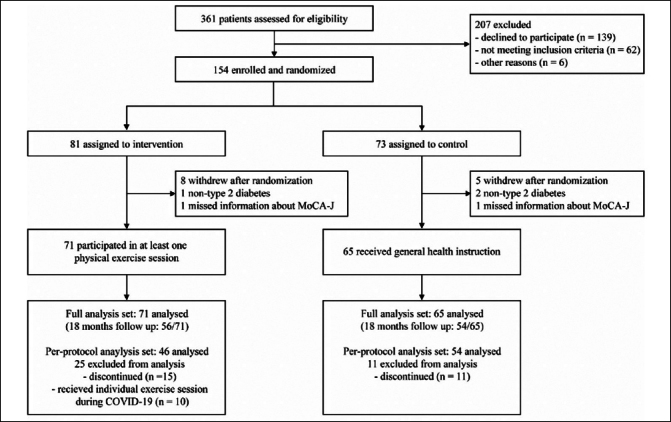
Trial profile MoCA-J, Japanese version of the Montreal Cognitive Assessment

Between March 13, 2019, and May 8, 2020, a total of 361 participants were screened. The COVID-19 pandemic started spreading in January 2020 in Japan and had an impact on recruitment and assessment. Because of the COVID-19 pandemic, we had to stop recruitment before reaching the target sample size. Out of the 361 participants, 154 participants were enrolled and randomly assigned to either the intervention group (n = 81) or the control group (n = 73) (Figure 1). The FAS included 136 participants after excluding 13 participants who withdrew immediately after randomization, three participants without type 2 diabetes, or two participants who lacked information about subitems of MoCA-J at baseline, which was necessary to assess eligibility (Figure 1). The baseline characteristics of the 136 participants were well-balanced (Table 1). Of the 136 participants included in the FAS, 26 discontinued the study for various reasons: COVID-19 pandemic (n = 13), physical illnesses such as musculoskeletal disorders and cardiac diseases (n = 5), and other personal reasons (n = 8). Finally, 110 participants (71%) completed the trial. Table S1 presents a comparison of baseline characteristics between individuals who completed and those who discontinued the trial. Participants who discontinued the trial were more likely to be older (P < 0.001) and have lower cognitive function in the Trail Making Test-Part A and Digit Symbol Substitution Test (P < 0.05).

**Table 1:** Baseline characteristics of the participants included in the full analysis set

	**Intervention group (n = 71)**	**Control group (n = 65)**	**P-value**
Sex			0.667
Male	43 (61%)	37 (57%)	
Female	28 (39%)	28 (43%)	
Age, years	77.0 (4.0)	76.9 (4.3)	0.896
Education, years	11.5 (2.3)	11.4 (2.5)	0.832
Height*, cm	157.8 (9.5)	157.8 (8.2)	0.961
Body weight*, kg	58.6 (10.7)	59.4 (11.3)	0.685
Body mass index*, kg/m2	23.5 (2.9)	23.8 (4.0)	0.539
Barthel index	99.4 (2.0)	99.5 (1.9)	0.746
HbA1c (%)	7.4 (1.0)	7.3 (0.8)	0.428
Glycemic control status			
Participants not receiving drugs potentially associated with a high risk of severe hypoglycemia (n = 68)			0.971
Within the target range (<7.0%)	20 (57%)	19 (58%)	
Above the upper limit (≥7.0%)	15 (43%)	14 (42%)	
Participants receiving drugs potentially associated with a high risk of severe hypoglycemia (n = 68)			0.671
Within the target range (7.0%–7.9%)	17 (47%)	16 (50%)	
Above the upper limit (≥8.0%)	13 (36%)	13 (41%)	
Below the lower limit (<7.0%)	6 (17%)	3 (9%)	
Use of insulin, sulfonylureas, and/or glinides	36 (51%)	32 (49%)	0.864
APOE ε4 carrier*	20/68 (29%)	14/62 (23%)	0.376
Composite score (mean Z score)*	0.03 (0.61)	−0.03 (0.59)	0.619
MoCA-J	20.3 (2.9)	20.3 (2.6)	0.952
MMSE	27.5 (2.2)	27.5 (2.0)	0.838
ROCFT			
Copy	30.0 (4.6)	29.7 (3.7)	0.700
Immediate recall	11.4 (5.8)	11.3 (6.3)	0.967
Delayed recall	10.4 (6.0)	10.4 (5.4)	0.983
Recall test of a 10-word list (errors)	3.6 (2.5)	3.7 (2.5)	0.815
Digit span			
Forward test	7.3 (1.9)	7.0 (2.0)	0.478
Backward test	4.6 (1.3)	4.3 (1.3)	0.253
Trail Making Test			
Part A*	70.9 (26.6)	69.8 (28.4)	0.824
Part B*	142.5 (65.1)	143.8 (62.9)	0.907
Digit symbol substitution test	43.1 (15.8)	41.8 (13.5)	0.588
Letter word fluency test	6.4 (2.4)	6.6 (2.4)	0.548

Data are presented as n (%), n/N (%), or mean (SD). *Data are not available for all randomized participants; HbA1c, glycated hemoglobin A1c; APOE, apolipoprotein E; MoCA-J, Japanese version of the Montreal Cognitive Assessment; MMSE, Mini-Mental State Examination; ROCFT, Rey-Osterrieth Complex Figure Test

### Interventional effects on cognitive outcomes

**Figure 2:**
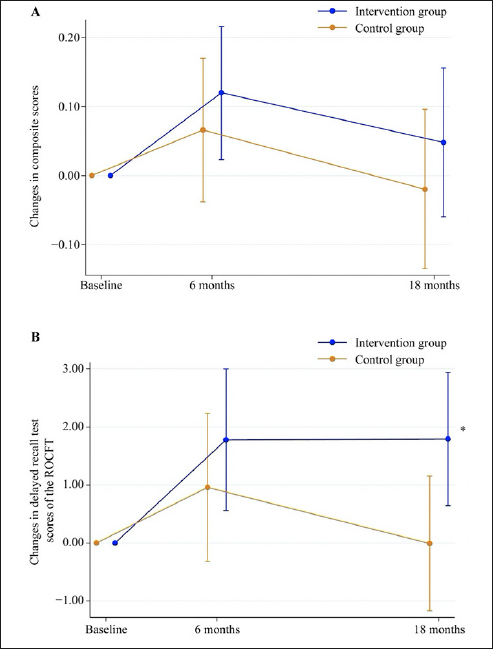
Changes in composite scores (A) and delayed recall test scores of the ROCFT (B) from baseline to the 18-month follow-up *Mean difference in changes in delayed recall test scores of the ROCFT between the intervention and control groups was statistically significant (P < 0.05). ROCFT, Rey-Osterrieth Complex Figure Test

In the primary analysis, the mean difference in the change of composite score from baseline to the 18-month follow-up between the intervention and control groups was not statistically significant (mean difference: 0.068, 95% confidence interval [CI]: −0.091 to 0.226, P = 0.398; Cohen’s d = 0.180; Table 2, Figure 2A). When analyzing each neuropsychological test, the mean difference in the change in delayed recall test score of the ROCFT between the groups was statistically significant (mean difference: 1.803, 95% CI: 0.169 to 3.436, P = 0.031; Cohen’s d = 0.404; Table 2, Figure 2B). The analysis using the PPS yielded similar results (Table S2).

**Table 2:** Estimated mean differences in changes in the composite cognitive score and neuropsychological tests from baseline to 6- and 18-month follow-up in the full analysis set

	**Follow-up**	**Intervention group (n = 71)**	**Control group (n = 65)**	**Mean differences between groups**	**P-value**
Composite score	6 months	0.120 (0.023 to 0.216)	0.066 (−0.038 to 0.170)	0.053 (−0.089 to 0.196)	0.459
	18 months	0.048 (−0.060 to 0.156)	−0.020 (−0.135 to 0.096)	0.068 (−0.091 to 0.226)	0.398
MoCA-J	6 months	0.443 (−0.283 to 1.168)	−0.108 (−0.860 to 0.643)	0.551 (−0.494 to 1.595)	0.298
	18 months	0.574 (−0.203 to 1.351)	0.043 (−0.752 to 0.838)	0.531 (−0.581 to 1.643)	0.346
MMSE	6 months	−0.187 (−0.697 to 0.323)	−0.428 (−0.956 to 0.101)	0.241 (−0.494 to 0.976)	0.517
	18 months	−0.169 (−0.678 to 0.340)	−0.697 (−1.217 to −0.177)	0.527 (−0.201 to 1.256)	0.154
ROCFT					
^Copy^	6 months	−1.362 (−2.369 to −0.355)	−1.192 (−2.247 to −0.137)	−0.170 (−1.629 to 1.289)	0.818
	18 months	−1.966 (−2.965 to −0.967)	−2.509 (−3.539 to −1.480)	0.544 (−0.892 to 1.979)	0.455
Immediate recall	6 months	2.817 (1.581 to 4.054)	−0.220 (−1.512 to 1.071)	3.038 (1.250 to 4.826)	0.001
	18 months	1.627 (0.297 to 2.956)	0.495 (−0.863 to 1.854)	1.132 (−0.770 to 3.033)	0.241
Delayed recall	6 months	1.778 (0.557 to 2.998)	0.957 (−0.317 to 2.231)	0.821 (−0.944 to 2.585)	0.359
	18 months	1.793 (0.645 to 2.941)	−0.009 (−1.171 to 1.153)	1.803 (0.169 to 3.436)	0.031
Recall test of a 10-word list	6 months	−0.546 (−1.019 to −0.072)	−0.484 (−0.974 to 0.007)	−0.062 (−0.744 to 0.621)	0.858
	18 months	−0.512 (−1.046 to 0.022)	−0.004 (−0.552 to 0.544)	−0.507 (−1.273 to 0.258)	0.192
Digit span					
Forward test	6 months	0.033 (−0.351 to 0.418)	−0.034 (−0.430 to 0.363)	0.067 (−0.486 to 0.620)	0.810
	18 months	0.132 (−0.223 to 0.487)	0.352 (−0.011 to 0.715)	−0.220 (−0.729 to 0.289)	0.393
Backward test	6 months	0.178 (−0.163 to 0.518)	0.106 (−0.245 to 0.457)	0.071 (−0.419 to 0.562)	0.773
	18 months	0.034 (−0.281 to 0.349)	0.151 (−0.170 to 0.472)	−0.117 (−0.569 to 0.335)	0.610
Trail Making Test					
Part A	6 months	−7.307 (−13.566 to −1.047)	−3.174 (−9.769 to 3.422)	−4.133 (−13.228 to 4.962)	0.370
	18 months	5.422 (−1.516 to 12.360)	10.447 (3.138 to 17.757)	−5.025 (−15.104 to 5.053)	0.325
Part B	6 months	−5.828 (−17.534 to 5.878)	7.720 (−4.853 to 20.294)	13.548 (−30.728 to 3.632)	0.121
	18 months	−9.425 (−21.531 to 2.681)	3.481 (−9.279 to 16.241)	−12.906 (−30.509 to 4.698)	0.149
Digit symbol substitution test	6 months	1.982 (0.334 to 3.631)	2.877 (1.165 to 4.590)	−0.895 (−3.278 to 1.488)	0.458
	18 months	1.552 (−0.209 to 3.311)	−0.673 (−2.475 to 1.128)	2.225 (−0.302 to 4.752)	0.084
Letter word fluency test	6 months	0.282 (−0.315 to 0.879)	0.078 (−0.541 to 0.698)	0.203 (−0.657 to 1.064)	0.641
	18 months	0.554 (−0.092 to 1.201)	0.320 (−0.338 to 0.979)	0.234 (−0.689 to 1.157)	0.617

MoCA-J, Japanese version of Montreal Cognitive Assessment; MMSE, Mini-Mental State Examination; ROCFT, Rey-Osterrieth Complex Figure Test

### Interventional effects on secondary outcomes

**Figure 3:**
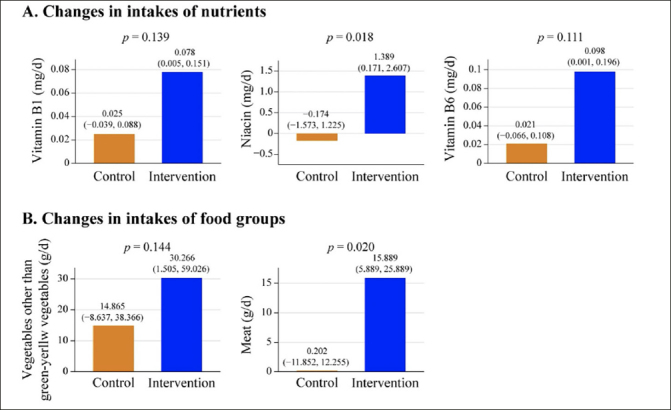
Changes in intakes of nutrients (A) and food groups (B) from baseline to 18-month follow-up The mean changes and the 95% confidence intervals within group were calculated using paired t-tests. The P-value for the mean differences between groups was calculated using the Wilcoxon rank sum test.

The analyses for secondary outcomes revealed that the intervention group showed the intakes of vitamin B1, niacin, vitamin B6, vegetables other than green-yellow vegetables, and meat increased within the intervention group. Specifically, there were statistically significant differences in the changes in niacin (P = 0.018) and meat (P = 0.020) intakes between the intervention and control groups (Figure 3, Table S3).

The intervention group exhibited a reduction in body weight and fat mass over the 18-month follow-up period, with a significant difference observed in the change in body weight between the intervention and control groups (P = 0.039) (Table S4, Figure S1). At the six-month follow-up, the intervention group demonstrated better performance in the one-leg standing test and a higher number of participating social groups compared to the control group, but these differences were not significant at the 18-month follow-up (Table S4, Figure S2 and S3). There were no significant interventional effects on other secondary outcomes, including diabetes-related outcomes and comprehensive geriatric assessment results (Tables S3-5).

### Subgroup analyses

Subgroup analyses based on glycemic control indicated that individuals in the intervention group with HbA1c levels outside the recommended target range experienced cognitive improvement (Table S6). However, the mean difference between the intervention and control groups was not statistically significant (mean difference: 0.102, 95% CI: −0.086 to 0.291). There were no significant differences in the effects of the intervention on the cognitive composite score between groups in terms of age at enrollment, use of drugs potentially associated with severe hypoglycemia, and APOE status (Table S6).

On average, each participant had a mean of 11.1 ± 8.9 exercise sessions canceled due to the COVID-19 pandemic. The impact of the pandemic was particularly significant after six months of the intervention, with participants attending an average of 9.3 ± 3.6 out of 13 sessions (72%) in the first six months, but only 11.0 ± 7.4 out of 26 sessions (42%) after six months. According to the analysis based on the attendance rate of group-based physical exercise sessions, the adherent intervention group (≥50%) demonstrated improvement in the composite score at six months. However, there were no significant differences in the composite score changes among the three groups at the six- and 18-month follow-up periods (Table S7, Figure S4).

### Post-hoc analyses

A post-hoc subgroup analysis based on sex revealed no significant intervention effect in males and females (Table S8). Furthermore, no significant intervention effect was observed among individuals with an MMSE score of >24 at enrollment (Table S8).

Post-hoc exploratory analyses (Supplemental Methods, page 7) revealed positive correlations between the increase in vitamin B1 and vitamin B6 intakes and changes in the cognitive composite score within the intervention group (Table S9). Furthermore, an increase in vitamin B1 and niacin intakes was also positively correlated with changes in the delayed recall test score of the ROCFT (Table S9).

### Serious adverse events

Of the 136 participants, 30 (22%) reported experiencing at least one SAE (Table S10). The most frequently reported events were related to eye disorders, particularly cataracts. The intervention group reported SAE more frequently than the control group (P = 0.009).

## Discussion

The J-MIND-Diabetes trial is the first study to investigate the efficacy of multidomain interventions in preventing cognitive decline in older adults with type 2 diabetes and cognitive impairment. Due to the COVID-19 pandemic, the trial only recruited approximately half of the intended sample size and faced limitations in providing interventions. Consequently, the multidomain interventions did not demonstrate significant efficacy in preventing cognitive decline. Nevertheless, further analyses of secondary outcomes suggested that the multidomain intervention had a potential benefit on memory and led to changes in participants’ dietary habits with increased intakes of niacin and meat, along with weight reduction over 18 months. Considering the abovementioned limitations, drawing firm conclusions regarding the efficacy of multidomain interventions in this study is challenging. However, this study offers proof-of-concept evidence for the use of multidomain interventions in older adults with type 2 diabetes and cognitive impairment.

To date, trials of multidomain interventions aimed at preventing cognitive decline in individuals with type 2 diabetes have been lacking. Furthermore, consistent with our findings, sub-analyses of the Look AHEAD trial did not demonstrate any intervention effect on cognitive decline (29, 30). A recent systematic review and meta-analysis indicated that multidomain interventions have limited beneficial effects on cognitive function, with stronger effects observed in populations at increased genetic risk for dementia (12). The AgeWell.de study, targeting individuals with a cardiovascular risk factors, aging, and dementia risk score of ≥9, did not demonstrate improvement in cognitive function as the primary outcome over a 24-month follow-up period (31). Similarly, the Japan-Multimodal Intervention Trial for the Prevention of Dementia study, targeting Japanese older adults with MCI, did not show significant intervention effects on the primary outcome over an 18-month follow-up period. However, this trial indicated that individuals with adequate adherence, APOE ε4 allele, or elevated plasma glial fibrillary acidic protein levels were more likely to benefit from interventions (32). The Systematic Multi-Domain Alzheimer Risk Reduction Trial, focusing on individuals with at least two of eight dementia risk factors, showed improvement in cognitive function over a 24-month follow-up period through personalized multidomain interventions, with poorly controlled diabetes evidenced either by hyperglycemia (HbA1c level of ≥8%) or hypoglycemia included as a risk factor (33). These findings highlight the importance of implementing intensive and individualized interventions tailored to specific populations. Indeed, although our study did not show significant differences between the two groups, potential benefits of cognitive function were observed in the intervention group among participants with HbA1c levels outside the recommended target range (Table S6). Further research is needed to identify target populations that are more likely to benefit from interventions in this population.

The COVID-19 pandemic had a significant impact on our trial. During the pandemic, the Japanese government strongly recommended staying at home, practicing physical distancing, and avoiding places with the “3Cs” (closed spaces, crowded places, and close-contact settings). In such a situation, group-based physical exercise sessions were restricted, especially after six months from the start of the trial. At the six-month follow-up, the intervention group showed better performance in the one-leg standing test and was more socially active compared to the control group, while these differences were not statistically significant at the 18-month follow-up. The World Health Organization recommends that older adults should engage in at least 150 min of moderate-to-vigorous exercise per week (34). Although we encouraged participants to engage in home-based exercise at least twice a week, the biweekly frequency of group-based exercise sessions may have been insufficient to achieve the full benefits of exercise.

Furthermore, this trial did not find any intervention benefits for glycemic control, as measured by HbA1c and CGM-derived metrics. This result may be attributed to the well-controlled glycemic status of the study population. The mean HbA1c level was 7.3%, and more than half of the participants maintained their glucose levels within the recommended target range at baseline. Moreover, the “Glycemic Targets for Elderly Patients with Diabetes” guidelines established by the JDS/JGS Joint Committee had a significant impact on diabetes treatment and clinical practices (16, 17), receiving widespread recognition. These findings suggest that patients in the control group were managed according to the guidelines, similar to those in the intervention group, making it challenging to detect a significant intervention effect on glycemic control. Although no improvement in metabolic control was noted, a potential benefit on memory was reported. An exploratory analysis conducted in the Lifestyle Interventions and Independence for Elders trial demonstrated that physical activity interventions promote cognitive function, particularly memory, in sedentary older adults with diabetes (35). Conversely, several trials focusing on the effects of intensive glycemic control could not provide evidence of its beneficial effects on cognitive function (36–38). These previous studies, and the current one, highlight the clinical significance of comprehensive diabetes management based on appropriate dietary habits and increased physical activity. This multidomain approach may contribute to better brain health in older adults with type 2 diabetes.

The importance of nutrition in the management of type 2 diabetes and its impact on weight and metabolic control are widely recognized. While changing long-term dietary habits in older people with diabetes can be challenging (39), analyses for secondary outcomes indicated that a substantial intervention, including face-to-face counseling every 2–3 months, the provision of study-provided placemats and foods, and self-monitoring using a dietary diary with feedback from dieticians, was effective in changing participants’ dietary habits and weight reduction. This intervention resulted in increased intakes of vitamin B1, niacin, vitamin B6, vegetables other than green-yellow vegetables, and meat. The study also found that increases in the intake of vitamin B1, niacin, and vitamin B6, were correlated with improvements in cognition among the intervention group. Previous studies have shown associations between niacin and B vitamin intake and the risk of cognitive decline and dementia in older adults (40–42). One study found significant correlations between niacin and vitamin B1 intake and memory, as measured by the ROCFT (40). Our findings are consistent with previous research, suggesting that changes in nutrient intake may contribute to improving memory, to some extent. However, our findings should be considered exploratory and future studies are required to confirm this.

In this trial, the intervention group reported a higher frequency of SAE than the control group. This is because SAE were assessed and recorded every 2 weeks in the intervention group, whereas they were only assessed and recorded at the six- and 18-month follow-ups in the control group.

This trial has a few limitations. First, our trial could not achieve the target sample size because of the COVID-19 pandemic, which may have resulted in low statistical power to detect the intervention effect and low accuracy of the estimate. Second, the COVID-19 pandemic had an impact on the provision of interventions, particularly group-based exercise. Several participants could not continue the trial due to factors associated with the COVID-19 pandemic. Additionally, those who discontinued the trial were older and had slightly lower cognitive function at enrollment, which may be linked to cognitive decline. These factors revealed that the intervention effect in our trial might have been underestimated. Third, in the current study, the control group received interventions such as standard diabetes care and general health-related information. They were also provided with all test results, except for cognitive function, which may have influenced their behavior. This study design may have posed challenges in detecting significant differences. Fourth, the durations of intervention and follow-up in our study were relatively shorter than those in trials that have demonstrated a significant intervention effect (10, 33). As the effect sizes of multidomain interventions may be small at individual and group levels over a short-term study period (12), longer intervention and follow-up trials are warranted. Fifth, the trial revealed significant differences in the changes observed in several secondary outcomes. However, notably, the significant results in the secondary outcome analysis were obtained through multiple statistical tests and were not considered confirmatory. Consequently, the control of statistical errors in these analyses may have been compromised, warranting further investigation in future studies. Sixth, this study lacked data on other clinical outcomes, such as dementia diagnosis and admission to nursing homes. Finally, our trial focused on Japanese older adults with type 2 diabetes and cognitive impairment. Even after excluding individuals with an MMSE score of <24, which is indicative of mild dementia, the results remained consistent. However, the generalizability of our findings to other populations, particularly those in primary prevention settings, may be limited.

In conclusion, the J-MIND-Diabetes trial did not show significant changes in the primary endpoints, possibly due to factors such as a small sample size, limited interventions, generally good glycemic status, and relatively shorter intervention and follow-up periods. However, this study provided proof-of-concept evidence that multidomain interventions could have benefits on memory, change dietary habits, and reduce weight in older adults with type 2 diabetes and cognitive impairment. Further research with larger sample sizes, targeting individuals with poorly managed diabetes, and longer intervention and follow-up periods could provide a more comprehensive understanding of the effectiveness of multidomain interventions in patients with type 2 diabetes.

## Supplemental Material

Supplementary material, approximately 1.08 MB.

## Notes

*Trial registration:* The trial was registered at the University Hospital Medical Information Network Clinical Trials Registry (UMIN-CTR) on February 18, 2019 (UMIN000035911). (https://center6.umin.ac.jp/cgi-open-bin/ctr_e/ctr_view.cgi?recptno=R000040908).

## Notes

*Ethical standards:* The study procedures were reviewed and approved by the Institutional Review Boards at all participating institutions. All participants provided written informed consent before participating in the trial.
