# Supplementary material for: Multidomain Intervention Trial for Preventing Cognitive Decline among Older Adults with Type 2 Diabetes: J-MIND-Diabetes
Source: J Prev Alzheimers Dis. 2024 Jun 26;11(6):1604–14. doi: 10.14283/jpad.2024.117 (PMC11573805; doi:10.14283/jpad.2024.117)
Supplement: Supplementary file 1 — Supplementary material, approximately 1.08 MB. [file 42414_2024_117_MOESM1_ESM.docx]

**Supplemental Material**

**Contents**

1. The J-MIND-Diabetes executive committee members
2. The J-MIND-Diabetes investigators
3. Supplemental methods
   1. Inclusion criteria
   2. Exclusion criteria
   3. Glycemic targets
   4. Details of secondary outcomes
      1. Diabetes-related outcomes
      2. Comprehensive geriatric assessment
   5. Post-hoc explanatory analysis of the association between changes in nutrient or food-group intakes and cognitive function changes among the intervention group
4. References
5. Baseline characteristics of the participants who completed and discontinued the trial in the full analysis set
6. Estimated mean differences in changes in the composite cognitive score and neuropsychological tests from baseline to 6- and 18-month follow-up in the per protocol set
7. Mean differences in changes in appetite and intakes of nutrients and food groups from baseline to 18-month follow-up in the full-analysis set
8. Mean differences in changes in secondary continuous outcomes from baseline to 6- and 18-month follow-up in the full-analysis set
9. Changes in secondary categorical outcomes from baseline to 6- and 18-month follow-up in the full-analysis set
10. Estimated mean differences in composite score changes from baseline to the 18-month follow-up among subgroups
11. Estimated mean differences in composite cognitive score changes from baseline to 6- and 18-month follow-up according to the attendance rate of group-based physical exercise sessions (attendance rate of 50% or greater, attendance rate less than 50%, and control group)
12. Estimated mean differences in composite score changes from baseline to the 18-month follow-up among subgroups (post-hoc analysis)
13. Association of changes in intakes of vitamin B1, niacin, vitamin B6, vegetables other than green-yellow vegetables, and meat with changes in the composite cognitive score and delayed recall test scores of the Rey–Osterrieth Complex Figure Test among the intervention group
14. Serious adverse events developed during the trial
15. Changes in body weight from baseline to 18-month follow-up
16. Changes in the performance of the one-leg standing test from baseline to 18-month follow-up
17. Changes in the number of participating social groups from baseline to 18-month follow-up
18. Changes in composite scores from baseline to 18-month follow-up based on the attendance rate of group-based physical exercise sessions (attendance rate of 50% or greater, attendance rate less than 50%, and control group)
19. **The J-MIND-Diabetes executive committee members**

Takashi Sakurai (Principal investigator, National Center for Geriatrics and Gerontology, Obu, Aichi, Japan); Atsushi Araki (Tokyo Metropolitan Institute for Geriatrics and Gerontology, Itabashi-Ku, Tokyo, Japan); Hiroki Fujita (Akita University Graduate School of Medicine, Akita, Japan), Keiko Honda (Kagawa Nutrition University, Sakado, Saitama, Japan); Nobuya Inagaki (Kyoto University Graduate School of Medicine, Kyoto, Japan); Takeshi Ishida (Tokyo Medical and Dental University, Tokyo, Japan); Junichi Kato (Hyogo Prefectural Rehabilitation Center at Nishi-Harima, Tatsuno, Hyogo); Minoru Kishi (Nishiwaki Municipal Hospital, Nishiwaki, Hyogo, Japan); Kazuki Kobayashi (Chiba Kaihin Municipal Hospital, Chiba, Japan); Kunichi Kouyama (Hyogo-Chuo National Hospital, Sanda, Hyogo, Japan); So Kuwahata (Tarumizu Municipal Medical Center, Tarumizu City, Kagoshima, Japan); Hisashi Noma (Institute of Statistical Mathematics, Tachikawa, Tokyo, Japan); Jun Ogino (Asahi General Hospital, Asahi, Chiba, Japan); Masahito Ogura (Kyoto University Graduate School of Medicine, Kyoto, Japan); Mitsuru Ohishi (Kagoshima University, Kagoshima, Japan); Kazuhiro Sugimoto (Ohta Nishinouchi Hospital, Koriyama, Fukushima, Japan); Taiki Sugimoto (National Center for Geriatrics and Gerontology, Obu, Aichi, Japan); Toshihiro Takenaka (Tarumizu Municipal Medical Center, Tarumizu City, Kagoshima, Japan); Yoshiaki Tamura (Tokyo Metropolitan Institute for Geriatrics and Gerontology, Itabashi-Ku, Tokyo, Japan); Haruhiko Tokuda (National Center for Geriatrics and Gerontology, Obu, Aichi, Japan); and Hiroyuki Umegaki (Nagoya University Graduate School of Medicine, Nagoya, Aichi, Japan).

1. **The J-MIND-Diabetes investigators**
2. National Center for Geriatrics and Gerontology: Takashi Sakurai, Taiki Sugimoto, Nanae Matsumoto, Kazuaki Uchida, Yoshinobu Kishino, Kosuke Fujita, Yujiro Kuroda, Takafumi Ando, Naoki Itoh, Masanori Tanimoto, Keita Aimoto, Saeko Omura, Fumi Fujii, Shuji Kawashima, Haruhiko Tokuda, Atsushi Maeda, Ai Kimura, and Hiroyuki Shimada
3. Tokyo Metropolitan Institute for Geriatrics and Gerontology: Atsuhi Araki, Yoshiaki Tamura, Yuko Chiba, Kenji Toyoshima, Kazuhito Oba, Remi Kodera, Yuji Murao, and Fumino Yorikawa
4. Akita University Graduate School of Medicine: Hiroki Fujita, Yukiko Toda, Mihoko Saito, and Yu Kume
5. Kagawa Nutrition University: Keiko Honda and Noriko Fukawa
6. Kyoto University Graduate School of Medicine: Nobuya Inagaki, Masahito Ogura, and Takaaki Murakami
7. Tokyo Medical and Dental University: Takeshi Ishida
8. Hyogo Prefectural Rehabilitation Center at Nishi-Harima: Junichi Kato
9. Nishiwaki Municipal Hospital: Minoru Kishi, Masatoshi Utaka, Yuri Oyama, Noriko Tominaga, and Reiko Yagami
10. Asahi General Hospital: Kazuki Kobayashi and Jun Ogino
11. Hyogo-Chuo National Hospital: Kunichi Kouyama, Michiru Fukunaga
12. Tarumizu Municipal Medical Center: So Kuwahata and Toshihiro Takenaka
13. Institute of Statistical Mathematics: Hisashi Noma
14. Kagoshima University: Mitsuru Oishi, Yoshiyuki Ikeda, and Yuichi Akasaki
15. Ohta Nishinouchi Hospital: Kazuhiro Sugimoto
16. Nagoya University Graduate School of Medicine: Hiroyuki Umegaki
17. **Supplemental methods**
    1. Inclusion criteria
18. Patients with type 2 diabetes
19. Patients aged 70–85 years at enrollment
20. Patients with no or mild impairment of basic activities of daily living, with a Barthel Index^1^ score of 80 or more
21. Patients with a Montreal Cognitive Assessment^2^ score of less than 26 (Japanese version)
22. Patients with a Mini-Mental State Examination^3^ score between 21 and 30
23. Outpatients or patients with a stable clinical course for more than 4 weeks since their last hospitalization or institutionalization, with no need for changes in their medical examination classification during the monitoring period
24. Patients accompanied by a coparticipant (study partner)
25. Patients who provided written informed consent
    1. Exclusion criteria
26. Patients with extremely poor metabolic control, as indicated by a fasting plasma glucose level over 250 mg/dL or moderate to severe urinary ketone levels
27. Patients with new hemorrhage in the ocular fundus caused by proliferative retinopathy
28. Patients with renal failure
29. Patients with ischemic heart disease and cardiopulmonary disorders
30. Patients with bone or joint disease
31. Patients with acute infectious disease
32. Patients with diabetic gangrene
33. Patients with severe autonomic neuropathy
34. Patients with decreased cognitive function due to Parkinson’s disease, apoplexy, Huntington’s disease, normal pressure hydrocephalus, brain tumors, progressive supranuclear palsy, corticobasal degeneration, multiple system atrophy, aphasia, epilepsy, subdural hematoma, encephalitis/meningitis, multiple sclerosis, or head injury
35. Patients with any local lesion, such as cerebral infarction(s) detected by magnetic resonance imaging (MRI) or computed tomography (CT) scan before enrollment, that may greatly affect cognitive function
36. Patients with a history of major depression, bipolar disorder, schizophrenia, or alcohol/drug abuse, or those with a current serious or unstable disease
37. Patients unsuitable for treatment due to vitamin B1, vitamin B12, and/or folate deficiency, syphilis, or thyroid dysfunction
38. Patients deemed ineligible for enrollment by their responsible investigator or coinvestigator at participating institutions
    1. Glycemic targets

Based on the “Glycemic Targets for Elderly Patients with Diabetes” by the JDS/JGS Joint Committee^4^, the participants’ glycemic control status in the trial was categorized into three groups: “within target range,” “above upper limit,” or “below lower limit.” This categorization was based on whether they were receiving drugs potentially associated with severe hypoglycemia, i.e., insulin, sulfonylureas, and/or glinides. For participants not receiving these drugs, the upper limit for glycated hemoglobin A1c (HbA1c) levels was set at 7.0%, while no specific lower limit was specified. For participants receiving these drugs, the upper limit for HbA1c levels was set at 8.0%, and the lower limit was set at 7.0%. Therefore, the target range for HbA1c levels in this trial was determined to be 7.0%–7.9%.

- 1. Detailed secondary outcomes
     1. Diabetes-related outcomes

1. Metabolic control: At baseline and 6- and 18-month follow-up, the participants were assessed for HbA1c and glycoalbumin levels. Additionally, at baseline and the 18-month follow-up, continuous glucose monitoring was conducted for up to 14 days using the FreeStyle Libre Pro system (Abbott Japan, Tokyo, Japan). This system can automatically measure glucose levels every 15 minutes without requiring scanning by the participants. The mean and standard deviation (SD) of the sensor glucose (SG) were calculated using the EasyGV Version 9.0 software.^5^ Various measures of glycemic variability were also calculated, including the glucose coefficient of variation (CV) as 100 × (SD)/mean SG, mean amplitude of glycemic excursions (MAGE), continuous overall net glycemic action (CONGA), and mean of daily differences (MODD).^6^ Additionally, the percentage of time spent in hypoglycemia (< 70 mg/dL) was calculated. For the analysis, data from patients with continuous glucose monitoring for 10 to 14 days were used.^7^
2. Self-reported hypoglycemic events: To assess hypoglycemic events, participants were asked the following questions: “Did you experience any hypoglycemia episode that required assistance from another person in the past year?” (Yes/No); “did you experience any symptoms such as sweating, palpitation, or trembling, in the past year?” (Yes/No); and “did you experience any symptoms such as lightheadedness, unsteadiness, dizziness, or visual disturbance in the past year?”
3. Microangiopathy and macroangiopathy: Participants were assessed for microangiopath, including retinopathy, nephropathy, and neuropathy, at baseline, 6-month follow-up (optional), and 18-month follow-up. Diabetic retinopathy was categorized into five stages:^8^ (1) no apparent retinopathy, (2) mild nonproliferative retinopathy, (3) moderate nonproliferative retinopathy, (4) severe nonproliferative retinopathy, and (5) proliferative diabetic retinopathy. Participants were also assessed for the presence or absence of macular edema. Diabetic nephropathy was categorized into five stages:^9^ (1) prenephropathy, (2) incipient nephropathy, (3) overt nephropathy, (4) kidney failure, and (5) dialysis therapy. Diabetic neuropathy was assessed using the Achilles tendon reflex test and vibration testing with a 128-Hz graduated tuning fork.^9^ The presence of cerebral hemorrhage and ischemic stroke was assessed based on the participants’ clinical charts, MRI, or CT scan. Furthermore, periventricular and deep white matter hyperintensities were assessed using the Fazekas scale.^10^
   - 1. Comprehensive geriatric assessment
4. Basic activities of daily living (ADL): Basic ADL was assessed using the Barthel index.^1^ This index includes 10 items that assess basic self-care abilities such as feeding, transferring from bed to chair, bathing, bowel control, and bladder control. The score ranges from 0 (complete dependence) to 100 (complete independence).
5. Instrumental ADL: Instrumental ADL was assessed using the Lawton index.^11^ This index includes eight items, with a higher total score (0–8) indicating greater independence in instrumental ADL. Of note, three items, i.e., “prepare food,” “housecleaning,” and “laundry,” were excluded from the calculation of the total score of the Lawton index for men.
6. Depressive symptoms: Depressive symptoms were assessed using the 15-item Geriatric Depression Scale.^12^ The score ranges from 0 to 15, with higher scores indicating a more depressed mood.
7. Anthropometric measurements: Height and body weight were measured, and body mass index was calculated by dividing body weight by the square of height (kg/m^2^). Additionally, fat mass (kg) and fat-free mass (kg) were assessed using bioelectrical impedance analysis.
8. Physical performance: Usual gait speed over a 2.4-meter distance was measured twice, and the mean value was calculated.^13^ Both right and left hand grip strengths were measured using a standard digital hand grip dynamometer (Takei Scientific Instruments Co., Ltd., Japan) at the standing position, with shoulders adducted and neutrally rotated, and elbows in full extension.^14^ The highest grip strength value was used in the analyses. The one-leg standing test was performed once for each leg with eyes open, in bare feet, for up to 60 seconds,^15^ and the longest time achieved in seconds was used in the analyses.
9. Physical frailty and sarcopenia: Physical frailty was defined based on the frailty phenotype proposed by Fried *et al*.^16^ in the Cardiovascular Health Study. This phenotype includes the following criteria: shrinking, weakness, slowness, self-reported exhaustion, and low physical activity. In addition, to determine the presence or absence of sarcopenia, appendicular muscle mass was measured using bioelectrical impedance analysis, and the skeletal muscle mass index was calculated by dividing appendicular muscle mass by squared height (kg/m^2^). Sarcopenia was defined as having a low skeletal muscle mass index, a low hand grip strength (men: <28 kg; women: <18 kg), and/or a slow gait speed (<1 m/s).^17,18^
10. Fall risk: Fall risk was assessed using the Fall Risk Index,^19^ which consists of 21 questions divided into three subcategories: physical function (8 items), geriatric syndrome (8 items), and environmental hazards (5 items). A higher score on this index indicates a higher risk of falling.
11. Nutritional status: Nutritional status was assessed using the Mini-Nutritional Assessment Short-Form,^20^ which includes six questions. The assessment score ranges from 0 to 14, with a higher score indicating better nutritional status.
12. Social network: The Lubben Social Network Scale-6^21^ was used to assess each participant’s social network. The scale score ranges from 0 to 30, with a higher score indicating better social network.
13. Social participation: Participants were assessed for their social participation using a questionnaire that asked about their social participation in eight types of groups:^22^ (1) neighborhood associations, senior citizen clubs, fire-fighting teams (Local Community); (2) hobby groups (Hobby); (3) sports groups or clubs (Sports); (4) political organizations or groups (Politics); (5) industrial or trade associations (Industry); (6) religious organizations or groups (Religion); (7) volunteer groups (Volunteer); and (8) Others. The number of participating groups that participants were involved in at least once per month was used in the analyses.
14. Number of medications: The number of medications prescribed to participants, excluding eye drops, nasal sprays, and topical medications, was assessed.
15. Appetite: At baseline and the 18-month follow-up, all participants were assessed for their appetite using the Simplified Nutritional Appetite Questionnaire.^23^ This questionnaire provides a total score ranging from 4 to 20, with a higher score indicating a better appetite.
16. Intakes of nutrients and food groups: All participants were assessed for their daily nutrient intakes and food-group consumption at baseline and the 18-month follow-up using the Food Frequency Questionnaire based on food groups.^24^
    1. Post-hoc explanatory analysis of the association between changes in nutrient or food-group intakes and cognitive function changes among the intervention group

The intervention group showed significant increases in the intakes of vitamin B1, niacin, vitamin B6, vegetables other than green-yellow vegetables, and meat. The differences in the changes in niacin (*p* = 0.018) and meat (*p* = 0.020) intakes from baseline to 18-month follow-up were significant between the intervention and control groups (Table S3). In the post-hoc explanatory analyses, to determine whether these changes in food-group and nutrient intakes contributed to cognitive improvement among the intervention group, multiple regression analyses were performed. The changes in the composite scores or delayed recall test scores of the Rey–Osterrieth Complex Figure Test were entered as dependent variables. The changes in intakes of vitamin B1, niacin, vitamin B6, vegetables other than green-yellow vegetables, or meat were entered as dependent variables. Furthermore, baseline age, sex, years of education, composite cognitive score, or delayed recall test scores of the Rey–Osterrieth Complex Figure Test were entered as covariates.

1. **References**
2. Mahoney FI, Barthel DW. Functional evaluation: The Barthel index. Md State Med J 1965;14:61-65.
3. Fujiwara Y, Suzuki H, Yasunaga M, et al. Brief screening tool for mild cognitive impairment in older Japanese: validation of the Japanese version of the Montreal Cognitive Assessment. Geriatr Gerontol Int 2010;10:225-232.
4. Folstein MF, Folstein SE, McHugh PR. “Mini-mental state.” A practical method for grading the cognitive state of patients for the clinician. J Psychiatr Res 1975;12:189-198.
5. Committee Report: Glycemic targets for elderly patients with diabetes: Japan Diabetes Society (JDS)/Japan Geriatrics Society (JGS) Joint Committee on Improving Care for Elderly Patients with Diabetes. J Diabetes Investig 2017;8:126-128.
6. Easy GV. <http://www.phc.ox.ac.uk/research/diabetes/software/easygv>. Accessed on 27 April 2023
7. Inchiostro S, Candido R, Cavalot F. How can we monitor glycemic variability in the clinical setting? Diabetes Obes Metab 2013;15:13-16.
8. Battelino T, Danne T, Bergenstal RM, et al. Clinical targets for continuous glucose monitoring data interpretation: recommendations from the international consensus on time in range. Diabetes Care 2019;42:1593-1603.
9. Wilkinson CP, Ferris FL 3rd, Klein RE, et al. Proposed international clinical diabetic retinopathy and diabetic macular edema disease severity scales. Ophthalmology 2003;110:1677-1682.
10. Japan Diabetes Society. Treatment guide for diabetes 2016-2017. Tokyo: Bunkodo, 2016:41-47.
11. Fazekas F, Chawluk JB, Alavi A, Hurtig HI, Zimmerman RA. MR signal abnormalities at 1.5 T in Alzheimer’s dementia and normal aging. AJR Am J Roentgenol 1987;149:351-356.
12. Lawton MP, Brody EM. Assessment of older people: self-maintaining and instrumental activities of daily living. Gerontologist. 1969;9:179-186.
13. Yesavage JA, Brink TL, Rose TL, et al. Development and validation of a geriatric depression screening scale: a preliminary report. J Psychiatr Res. 1982-1983;17:37-49.
14. Guralnik JM, Simonsick EM, Ferrucci L, et al. A short physical performance battery assessing lower extremity function: association with self-reported disability and prediction of mortality and nursing home admission. J Gerontol 1994;49:M85-94.
15. Watanabe T, Owashi K, Kanauchi Y, Mura N, Takahara M, Ogino T. The short-term reliability of grip strength measurement and the effects of posture and grip span. J Hand Surg Am 2005;30:603-609.
16. Michikawa T, Nishiwaki Y, Takebayashi T, Toyama Y. One-leg standing test for elderly populations. J Orthop Sci 2009;14:675-685.
17. Fried LP, Tangen CM, Walston J, et al. Frailty in older adults: evidence for a phenotype. J Gerontol A Biol Sci Med Sci 2001;56:M146-156.
18. Cruz-Jentoft AJ, Bahat G, Bauer J, et al. Sarcopenia: revised European consensus on definition and diagnosis. Age Ageing 2019;48:16-31.
19. Chen LK, Woo J, Assantachai P, et al. Asian Working Group for Sarcopenia: 2019 Consensus Update on Sarcopenia Diagnosis and Treatment. J Am Med Dir Assoc 2019;21:300-307.e2.
20. Kikuchi R, Kozaki K, Iwata A, Hasegawa H, Toba K. Evaluation of risk of falls in patients at a memory impairment outpatient clinic. Geriatr Gerontol Int 2009;9:298-303.
21. Rubenstein LZ, Harker JO, Salvà A, Guigoz Y, Vellas B. Screening for undernutrition in geriatric practice: developing the short-form mini-nutritional assessment (MNA-SF). J Gerontol A Biol Sci Med Sci 2001;56:M366-372.
22. Lubben J, Blozik E, Gillmann G, et al. Performance of an abbreviated version of the Lubben Social Network Scale among three European Community-dwelling older adult populations. Gerontologist 2006;46:503-513.
23. Kanamori S, Kai Y, Aida J, et al. Social participation and the prevention of functional disability in older Japanese: the JAGES cohort study. PLoS One 2014;9:e99638.
24. Nakatsu N, Sawa R, Misu S, Ueda Y, Ono R. Reliability and validity of the Japanese version of the simplified nutritional appetite questionnaire in community-dwelling older adults. Geriatr Gerontol Int 2015;15:1264-1269.
25. Sasaki S, Kobayashi M, Tsugane S. Validity of a self-administered food frequency questionnaire used in the 5-year follow-up survey of the JPHC Study Cohort I: comparison with dietary records for food groups. J Epidemiol 2003;13, S57-63.

**Table S1. Baseline characteristics of the participants who completed and discontinued the trial in the full analysis set**

|  | **Discontinued (*n* = 26)** | **Completed (*n* =110)** | **P-value** |
| --- | --- | --- | --- |
| Sex |  |  | 0.231 |
| Male | 18 (69%) | 62 (56%) |  |
| Female | 8 (31%) | 48 (44%) |  |
| Age, years | 79.5 (3.3) | 76.3 (4.1) | <0.001 |
| Education, years | 11.5 (2.4) | 11.5 (2.5) | 0.986 |
| Height^*^, cm | 159.4 (8.4) | 157.4 (9.0) | 0.313 |
| Body weight^*^, kg | 59.6 (11.1) | 58.8 (11.0) | 0.743 |
| Body mass index^*^, kg/m^2^ | 23.4 (3.3) | 23.7 (3.5) | 0.691 |
| Barthel index | 100.0 (0.0) | 99.4 (2.2) | 0.133 |
| HbA1c (%) | 7.3 (1.2) | 7.3 (0.8) | 0.944 |
| Glycemic control status |  |  |  |
| Participants not receiving drugs potentially associated with a high risk of severe hypoglycemia (*n* = 68) |  |  | 0.721 |
| Within the target range (<7.0%) | 8 (53%) | 31 (58%) |  |
| Above the upper limit (≥ 7.0%) | 7 (47%) | 22 (42%) |  |
| Participants receiving drugs potentially associated with a high risk of severe hypoglycemia (*n* = 68) |  |  | 0.476 |
| Within the target range (7.0%–7.9%) | 4 (36%) | 29 (51%) |  |
| Above the upper limit (≥ 8.0%) | 6 (55%) | 20 (35%) |  |
| Below the lower limit (<7.0%) | 1 (9%) | 8 (14%) |  |
| Use of insulin, sulfonylureas, and/or glinides | 11 (42%) | 57 (52%) | 0.383 |
| APOE ε4 carrier^*^ | 3 (12%) | 31 (30%) | 0.073 |
| Composite score (mean Z score)^*^ | −0.12 (0.52) | 0.03 (0.62) | 0.252 |
| MoCA-J | 19.8 (2.9) | 20.5 (2.7) | 0.257 |
| MMSE | 27.3 (2.0) | 27.6 (2.1) | 0.533 |
| ROCFT |  |  |  |
| Copy | 29.1 (3.9) | 30.0 (4.3) | 0.306 |
| Immediate recall | 10.5 (5.2) | 11.5 (6.2) | 0.444 |
| Delayed recall | 8.4 (5.5) | 10.9 (5.7) | 0.051 |
| Recall test of a 10-word list (errors) | 3.7 (2.3) | 3.6 (2.6) | 0.975 |
| Digit span |  |  |  |
| Forward | 7.0 (1.5) | 7.2 (2.0) | 0.605 |
| Backward | 4.4 (1.1) | 4.5 (1.3) | 0.756 |
| Trail Making Test |  |  |  |
| Part A^*^ | 81.4 (19.0) | 67.9 (28.4) | 0.029 |
| Part B^*^ | 148.1 (56.5) | 141.9 (65.6) | 0.669 |
| Digit symbol substitution test | 37.3 (11.9) | 43.7 (15.1) | 0.044 |
| Letter word fluency test | 7.0 (2.4) | 6.5 (2.6) | 0.317 |

Data are presented as *n* (%), *n/N* (%), or mean (SD). ^*^Data are not available for all randomized participants. P-values were calculated using the t-test or Pearson chi-squared test.

HbA1c, glycated hemoglobin A1c; APOE, apolipoprotein E; MoCA-J, Japanese version of the Montreal Cognitive Assessment; MMSE, Mini-Mental State Examination; ROCFT, Rey–Osterrieth Complex Figure Test

**Table S2.** **Estimated mean differences in changes in the composite cognitive score and neuropsychological tests from baseline to 6- and 18-month follow-up in the per protocol set**

|  | **Follow-up** | **Intervention group (*n* = 46)** | **Control group (*n* = 54)** | **Mean differences between groups** | **P-value** |
| --- | --- | --- | --- | --- | --- |
| Composite score | 6 months | 0.102 (−0.000 to 0.203) | 0.052 (−0.050 to 0.154) | 0.050 (−0.096 to 0.195) | 0.498 |
|  | 18 months | −0.001 (−0.116 to 0.113) | −0.034 (−0.147 to 0.079) | 0.033 (−0.130 to 0.195) | 0.689 |
| MoCA-J | 6 months | 0.821 (0.015 to 1.627) | −0.268 (−1.009 to 0.473) | 1.089 (−0.006 to 2.184) | 0.051 |
|  | 18 months | 0.722 (−0.166 to 1.611) | −0.055 (−0.874 to 0.765) | 0.777 (−0.433 to 1.987) | 0.206 |
| MMSE | 6 months | −0.201 (−0.777 to 0.375) | −0.463 (−0.992 to 0.066) | 0.262 (−0.525 to 1.048) | 0.511 |
|  | 18 months | −0.193 (−0.764 to 0.377) | −0.719 (−1.244 to −0.193) | 0.525 (−0.254 to 1.305) | 0.184 |
| ROCFT |  |  |  |  |  |
| Copy | 6 months | −1.991 (−3.018 to −0.965) | −1.138 (−2.089 to −0.187) | −0.854 (−2.263 to 0.555) | 0.232 |
|  | 18 months | −2.831 (−3.883 to −1.780) | −2.500 (−3.479 to −1.520) | −0.331 (−1.777 to 1.114) | 0.650 |
| Immediate recall | 6 months | 3.205 (1.795 to 4.615) | −0.473 (−1.779 to 0.834) | 3.678 (1.754 to 5.602) | <0.001 |
|  | 18 months | 1.700 (0.221 to 3.179) | 0.354 (−1.009 to 1.717) | 1.346 (−0.668 to 3.359) | 0.188 |
| Delayed recall | 6 months | 2.218 (0.810 to 3.626) | 0.743 (−0.560 to 2.044) | 1.476 (−0.444 to 3.395) | 0.130 |
|  | 18 months | 2.151 (0.833 to 3.470) | −0.116 (−1.318 to 1.085) | 2.268 (0.482 to 4.054) | 0.013 |
| Recall test of a 10-word list | 6 months | −0.822 (−1.372 to −0.271) | −0.479 (−0.986 to 0.027) | −0.342 (−1.091 to 0.406) | 0.366 |
|  | 18 months | −0.749 (−1.361 to −0.137) | −0.005 (−0.570 to 0.560) | −0.744 (−1.578 to 0.090) | 0.080 |
| Digit span |  |  |  |  |  |
| Forward test | 6 months | −0.092 (−0.527 to 0.343) | −0.067 (−0.467 to 0.333) | −0.025 (−0.618 to 0.568) | 0.934 |
|  | 18 months | 0.079 (−0.312 to 0.470) | 0.325 (−0.036 to 0.686) | −0.246 (−0.780 to 0.288) | 0.363 |
| Backward test | 6 months | 0.339 (−0.038 to 0.716) | −0.024 (0.370 to 0.322) | 0.363 (−0.152 to 0.879) | 0.165 |
|  | 18 months | 0.040 (−0.310 to 0.389) | 0.087 (−0.235 to 0.409) | −0.047 (−0.526 to 0.431) | 0.845 |
| Trail Making Test |  |  |  |  |  |
| Part A | 6 months | −9.489 (−16.425 to −2.552) | −2.211 (−8.945 to 4.523) | −7.277 (−16.959 to 2.404) | 0.139 |
|  | 18 months | 6.162 (−1.529 to 13.853) | 11.173 (3.639 to 18.707) | −5.011 (−15.784 to 5.763) | 0.358 |
| Part B | 6 months | −4.679 (−17.670 to 8.312) | 8.185 (−4.711 to 21.082) | −12.864 (−31.230 to 5.501) | 0.167 |
|  | 18 months | −8.971 (−22.315 to 4.373) | 4.247 (−8.806 to 17.301) | −13.219 (−31.966 to 5.529) | 0.165 |
| Digit symbol substitution test | 6 months | 1.300 (−0.565 to 3.166) | 2.881 (1.169 to 4.592) | −1.580 (−4.141 to 0.980) | 0.224 |
|  | 18 months | 0.812 (−1.210 to 2.834) | −0.683 (−2.550 to 1.184) | 1.495 (−1.284 to 4.273) | 0.288 |
| Letter word fluency test | 6 months | 0.407 (−0.311 to 1.125) | −0.022 (−0.681 to 0.638) | 0.429 (−0.546 to 1.403) | 0.385 |
|  | 18 months | 0.546 (−0.168 to 1.259) | 0.248 (−0.410 to 0.907) | 0.297 (−0.674 to 1.268) | 0.545 |

MoCA-J, Japanese version of Montreal Cognitive Assessment; MMSE, Mini-Mental State Examination; ROCFT, Rey–Osterrieth Complex Figure Test

**Table S3. Mean differences in changes in appetite and intakes of nutrients and food groups from baseline to 18-month follow-up in the full-analysis set**

|  | **Baseline** | |  | **Changes from baseline to the 18-month follow-up** | | | |
| --- | --- | --- | --- | --- | --- | --- | --- |
|  | **Intervention group** | **Control group** |  | **Intervention group** | **Control group** | **Mean differences between groups** | **P*-*value** |
| **SNAQ** | 15.162 (14.722) | 15.516 (1.156) |  | 0.154 (−0.328 to 0.636) | −0.151 (−0.561 to 0.259) | 0.305 (−0.319 to 0.929) | 0.098 |
| **Nutritional intake** |  |  |  |  |  |  |  |
| Energy (Kcal/d) | 1692.600 (530.792) | 1673.930 (383.505) |  | 7.000 (−86.684 to 100.700) | −35.430 (−136.5 to 65.681) | 42.430 (−94.256 to 179.1) | 0.511 |
| Protein (g/d) | 62.522 (16.042) | 63.285 (17.58) |  | 33.538 (−12.336 to 79.413) | 14.175 (−14.699 to 43.050) | 19.363 (−33.828 to 72.554) | 0.148 |
| Fat (g/d) | 51.325 (15.044) | 51.273 (15.713) |  | 2.709 (−1.262 to 6.680) | 0.707 (−3.092 to 4.506) | 2.002 (−3.430 to 7.434) | 0.247 |
| Monounsaturated fatty acids (g/d) | 17.650 (5.267) | 18.182 (5.771) |  | 1.087 (−0.354 to 2.529) | 0.009 (−1.488 to 1.506) | 1.079 (−0.979 to 3.136) | 0.169 |
| Polyunsaturated fatty acids (g/d) | 11.012 (3.323) | 10.813 (3.411) |  | 0.544 (−0.504 to 1.592) | −0.177 (−1.125 to 0.770) | 0.721 (−0.674 to 2.116) | 0.146 |
| n-3 fatty acids (g/d) | 2.172 (0.776) | 2.066 (0.699) |  | 0.067 (−0.164 to 0.298) | −0.042 (−0.268 to 0.184) | 0.109 (−0.210 to 0.429) | 0.158 |
| n-6 fatty acids (g/d) | 8.809 (2.678) | 8.721 (2.877) |  | 0.476 (−0.371 to 1.324) | −0.130 (−0.920 to 0.660) | 0.606 (−0.539 to 1.751) | 0.183 |
| Cholesterol (mg/d) | 308.348 (117.762) | 322.668 (130.208) |  | 19.304 (−12.155 to 50.762) | 14.077 (−17.607 to 45.761) | 5.226 (−38.946 to 49.399) | 0.848 |
| Carbohydrate (g/d) | 233.053 (53.707) | 228.819 (59.112) |  | −18.027 (−35.419 to 0.636) | −12.640 (−30.105 to 4.825) | −5.387 (−29.770 to 18.996) | 0.568 |
| Na (mg/d) | 3615.87 (1420.760) | 3337.010 (1049.700) |  | 118.8 (−212.0 to 449.7) | 87.023 (−212.0 to 386.0) | 31.819 (−408.5 to 472.1) | 0.675 |
| K (mg/d) | 2241.130 (721.493) | 2223.370 (652.863) |  | 118.100 (−69.919 to 306.100) | 32.907 (−126.6 to 192.4) | 85.198 (−158.0 to 328.4) | 0.358 |
| Ca (mg/d) | 528.245 (184.163) | 467.285 (160.202) |  | −19.344 (−63.989 to 25.302) | 27.370 (−8.252 to 62.992) | 46.714 (−103.0 to 9.554) | 0.233 |
| Mg (mg/d) | 243.256 (71.373) | 236.803 (66.687) |  | 4.726 (−12.578 to 22.029) | −3.704 (−20.170 to 12.763) | 8.429 (−15.178 to 32.036) | 0.453 |
| P (mg/d) | 945.255 (249.763) | 936.339 (258.497) |  | 22.856 (−36.771 to 82.484) | 5.730 (−56.110 to 67.570) | 17.127 (−67.908 to 102.2) | 0.478 |
| Fe (mg/d) | 6.961 (2.039) | 6.826 (1.831) |  | 0.227 (−0.277 to 0.731) | −0.005 (−0.439 to 0.428) | 0.233 (−0.423 to 0.888) | 0.496 |
| Zn (mg/d) | 7.036 (1.620) | 7.247 (1.847) |  | 0.211 (−0.261 to 0.682) | −0.074 (−0.565 to 0.418) | 0.285 (−0.390 to 0.959) | 0.213 |
| Cu (mg/d) | 1.038 (0.246) | 1.034 (0.257) |  | 0.002 (−0.072 to 0.075) | −0.037 (−0.107 to 0.033) | 0.039 (−0.062 to 0.139) | 0.259 |
| Mn (mg/d) | 2.490 (0.604) | 2.453 (0.600) |  | −0.029 (−0.210 to 0.152) | −0.088 (−0.263 to 0.087) | 0.059 (−0.190 to 0.307) | 0.531 |
| Iodine (μg/d) | 794.863 (595.142) | 819.939 (585.642) |  | −60.718 (−271.8 to 150.3) | −130.8 (−304.2 to 42.626) | 70.075 (−199.2 to 339.3) | 0.894 |
| Se (μg/d) | 60.294 (19.603) | 57.869 (18.020) |  | 2.782 (−1.683 to 7.247) | 0.949 (−3.613 to 5.511) | 1.833 (−4.485 to 8.150) | 0.170 |
| Cr (μg/d) | 5.747 (1.934) | 5.615 (1.766) |  | 0.244 (−0.328 to 0.815) | −0.037 (−0.532 to 0.459) | 0.281 (−0.466 to 1.027) | 0.413 |
| Mo (μg/d) | 152.128 (43.970) | 152.834 (45.759) |  | −7.240 (−19.887 to 5.407) | −9.207 (−21.778 to 3.364) | 1.967 (−15.671 to 19.605) | 0.658 |
| Retinol equivalent (μg/d) | 519.056 (237.851) | 460.434 (169.405) |  | −6.289 (−69.777 to 57.199) | 34.212 (−9.317 to 77.741) | −40.501 (−116.1 to 35.189) | 0.537 |
| Vitamin D (μg/d) | 6.706 (3.393) | 6.263 (3.196) |  | 0.176 (−0.605 to 0.958) | 0.088 (−0.931 to 1.106) | 0.089 (−1.187 to 1.365) | 0.285 |
| αTocopherol (mg/d) | 6.780 (2.096) | 6.437 (1.817) |  | 0.226 (−0.359 to 0.809) | 0.060 (−0.430 to 0.549) | 0.166 (−0.585 to 0.917) | 0.568 |
| Vitamin K (μg/d) | 246.803 (120.250) | 224.742 (82.687) |  | 4.224 (−26.014 to 34.461) | 7.440 (−17.120 to 32.000) | −3.217 (−41.606 to 35.172) | 0.995 |
| Vitamin B1 (mg/d) | 0.870 (0.258) | 0.881 (0.257) |  | 0.078 (0.005 to 0.151) | 0.025 (−0.039 to 0.088) | 0.054 (−0.042 to 0.149) | 0.139 |
| Vitamin B2 (mg/d) | 1.114 (0.340) | 1.048 (0.324) |  | 0.011 (−0.073 to 0.095) | 0.054 (−0.013 to 0.122) | −0.044 (−0.150 to 0.063) | 0.627 |
| Niacin (mg/d) | 13.502 (4.629) | 14.277 (4.735) |  | 1.389 (0.171 to 2.607) | −0.174 (−1.573 to 1.225) | 1.563 (−0.277 to 3.402) | 0.018 |
| Vitamin B6 (mg/d) | 1.088 (0.348) | 1.131 (0.341) |  | 0.098 (0.001 to 0.196) | 0.021 (−0.066 to 0.108) | 0.077 (−0.052 to 0.206) | 0.111 |
| Vitamin B12 (μg/d) | 6.617 (3.139) | 6.400 (2.987) |  | 0.146 (−0.508 to 0.799) | −0.198 (−1.144 to 0.747) | 0.344 (−0.801 to 1.488) | 0.115 |
| Folic acid (μg/d) | 293.358 (116.326) | 281.308 (90.917) |  | 17.147 (−13.897 to 48.191) | 11.504 (−12.303 to 35.310) | 5.644 (−32.870 to 44.158) | 0.441 |
| Pantothenic acid (mg/d) | 5.164 (1.315) | 5.170 (1.416) |  | 0.160 (−0.181 to 0.501) | 0.132 (−0.258 to 0.468) | 0.028 (−0.445 to 0.502) | 0.566 |
| Biotin (μg/d) | 28.653 (8.472) | 28.892 (9.182) |  | 1.335 (−1.004 to 3.673) | 1.207 (−0.932 to 3.347) | 0.128 (−3.003 to 3.258) | 0.736 |
| Vitamin C (mg/d) | 86.947 (42.628) | 86.373 (36.271) |  | 8.360 (−4.526 to 21.246) | 4.565 (−4.541 to 13.671) | 3.795 (−11.717 to 19.307) | 0.370 |
| Soluble fiber (g/d) | 3.061 (1.035) | 2.879 (0.925) |  | 0.167 (−0.153 to 0.488) | 0.083 (−0.167 to 0.332) | 0.085 (−0.315 to 0.485) | 0.600 |
| Insoluble fiber (g/d) | 9.411 (3.230) | 9.102 (2.721) |  | 0.536 (−0.419 to 1.492) | 0.197 (−0.541 to 0.934) | 0.340 (−0.848 to 1.528) | 0.383 |
| Total fiber (g/d) | 13.331 (4.487) | 12.806 (3.801) |  | 0.595 (−0.709 to 1.898) | 0.197 (−0.826 to 1.219) | 0.398 (−1.234 to 2.030) | 0.490 |
| NaCl (g/d) | 9.145 (3.613) | 8.418 (2.661) |  | 0.286 (−0.556 to 1.127) | 0.228 (−0.532 to 0.988) | 0.057 (−1.062 to 1.177) | 0.707 |
| Ethanol (g/d) | 4.978 (9.334) | 4.223 (9.037) |  | 0.371 (−1.492 to 2.234) | 0.411 (−1.624 to 2.445) | −0.040 (−2.772 to 2.693) | 0.723 |
| **Intake of food groups** |  |  |  |  |  |  |  |
| Grains (g/d) | 371.622 (107.205) | 356.632 (115.186) |  | −30.176 (−60.206 to −0.147) | −27.421 (−58.184 to 3.341) | −2.755 (−45.300 to 39.789) | 0.665 |
| Potatoes (g/d) | 27.120 (26.051) | 34.156 (35.976) |  | −0.058 (−9.313 to 9.196) | −4.690 (−14.534 to 5.155) | 4.631 (−8.749 to 18.012) | 0.475 |
| Green-yellow vegetables (g/d) | 93.439 (65.150) | 75.832 (45.336) |  | −1.329 (−20.211 to 17.553) | 7.456 (−6.083 to 20.996) | −8.785 (−31.633 to 14.062) | 0.841 |
| Other vegetables (g/d) | 158.752 (100.988) | 158.603 (78.699) |  | 30.266 (1.505 to 59.026) | 14.865 (−8.637 to 38.366) | 15.401 (−21.205 to 52.006) | 0.144 |
| Seaweeds (g/d) | 4.253 (3.611) | 4.484 (3.519) |  | −0.247 (−1.547 to 1.053) | −0.721 (−1.749 to 0.307) | 0.474 (−1.159 to 2.106) | 0.977 |
| Soybeans/Soy products (g/d) | 59.250 (47.646) | 55.919 (39.964) |  | −0.127 (−11.912 to 11.657) | −5.211 (−17.160 to 6.739) | 5.083 (−11.523 to 21.689) | 0.297 |
| Fish/Seafoods (g/d) | 71.069 (45.700) | 67.598 (40.478) |  | 3.269 (−7.222 to 13.761) | −2.947 (−17.706 to 11.811) | 6.217 (−11.804 to 24.237) | 0.074 |
| Meat (g/d) | 48.483 (32.235) | 67.002 (39.440) |  | 15.889 (5.889 to 25.889) | 0.202 (−11.852 to 12.255) | 15.687 (0.142 to 31.232) | 0.020 |
| Eggs (g/d) | 34.928 (21.558) | 37.377 (22. 535) |  | 3.249 (−3.349 to 9.847) | 3.077 (−2.924 to 9.079) | 0.172 (−8.636 to 8.980) | 0.895 |
| Milk/Dairy products (g/d) | 157.286 (120.127) | 131.505 (92.223) |  | −21.476 (−51.300 to 8.348) | 18.209 (−7.833 to 44.250) | −39.685 (−78.754 to −0.617) | 0.164 |
| Fruits (g/d) | 104.460 (75.116) | 109.558 (101.601) |  | 15.195 (−13.162 to 43.551) | 10.528 (−22.216 to 43.272) | 4.667 (−38.295 to 47.628) | 0.991 |
| Snacks (g/d) | 39.292 (35.092) | 40.123 (34.115) |  | −2.995 (−12.308 to 6.319) | 0.479 (−10.318 to 11.276) | −3.474 (−17.617 to 10.670) | 0.942 |
| Sugar-sweetened beverages (g/d) | 84.036 (127.356) | 71.071 (123.600) |  | −2.291 (−32.148 to 27.566) | −3.432 (−34.871 to 28.008) | 1.141 (−41.790 to 44.071) | 0.903 |
| Sugar (g/d) | 8.366 (6.952) | 9.539 (7.007) |  | 1.204 (−0.682 to 3.089) | 0.119 (−1.374 to 1.613) | 1.084 (−1.285 to 3.453) | 0.633 |
| Nuts and seeds (g/d) | 2.861 (4.712) | 4.223 (8.084) |  | −0.135 (−1.334 to 1.065) | −0.205 (−1.866 to 1.456) | 0.071 (−1.968 to 2.109) | 0.825 |
| Fats and oils (g/d) | 12.750 (8.119) | 11.919 (6.795) |  | −0.867 (−3.480 to 1.746) | −0.135 (−2.351 to 2.081) | −0.732 (−4.111 to 2.647) | 0.861 |
| Seasonings and spices (g/d) | 19.131 (9.233) | 19.110 (10.127) |  | 1.744 (−0.554 to 4.042) | 0.984 (−1.850 to 3.818) | 0.759 (−2.863 to 4.382) | 0.391 |

The 95% confidence intervals for the mean changes within group and the mean differences between groups were calculated using paired and unpaired *t*-tests, respectively. The *p-*value for the mean differences between groups was calculated using the Wilcoxon rank sum test.

SNAQ, simplified nutritional appetite questionnaire

**Table S4. Mean differences in changes in secondary continuous outcomes from baseline to 6- and 18-month follow-up in the full-analysis set**

|  | **Follow-up** | **Intervention group** | **Control group** | **Mean differences between groups** | **P*-*value** |
| --- | --- | --- | --- | --- | --- |
| **Metabolic control** |  |  |  |  |  |
| HbA1c | 6 months | −0.061 (−0.277 to 0.154) | −0.066 (−0.277 to 0.154) | 0.005 (−0.243 to 0.252) | 0.583 |
|  | 18 months | −0.181 (−0.412 to 0.051) | 0.047 (−0.222 to 0.317) | −0.228 (−0.575 to 0.120) | 0.122 |
| Glycated albumin | 6 months | −0.503 (−1.255 to 0.249) | −0.356 (−1.050 to 0.337) | −0.147 (−1.147 to 0.853) | 0.871 |
|  | 18 months | −0.881 (−1.995 to 0.234) | −0.206 (−1.126 to 0.713) | −0.674 (−2.086 to 0.737) | 0.234 |
| **Metrics derived from CGM** |  |  |  |  |  |
| Sensor glucose | 18 months | 0.388 (-0.164 to 0.941) | 0.234 (-0.289 to 0.758) | 0.154 (-0.600 to 0.908) | 0.637 |
| Standard deviation | 18 months | 0.065 (-0.127 to 0.257) | −0.059 (-01.99 to 0.081) | 0.124 (-0.115 to 0.364) | 0.126 |
| %CV | 18 months | −0.265 (-1.986 to 1.456) | −1.710 (-3.700 to 0.280) | 1.445 (-1.123 to 4.013) | 0.484 |
| MAGE | 18 months | 0.265 (-0.145 to 0.675) | 0.144 (-0.259 to 0.547) | 0.121 (-0.447 to 0.689) | 0.436 |
| CONGA | 18 months | 0.365 (-0.154 to 0.883) | 0.183 (-0.283 to 0.649) | 0.182 (-0.512 to 0.875) | 0.570 |
| MODD | 18 months | 0.037 (-0.166 to 0.240) | −0.031 (-0.187 to 0.125) | 0.068 (-0.188 to 0.324) | 0.382 |
| Percentage time spent in hypoglycemia (<70 mg /dL) | 18 months | 0.435 (-2.086 to 2.957) | −2.026 (-5.000 to 0.949) | 2.461 (-1.340 to 6.262) | 0.286 |
| **Comprehensive geriatric assessment** |  |  |  |  |  |
| Barthel Index | 6 months | −0.263 (−1.248 to 0.721) | −0.196 (−0.823 to 0.430) | −0.067 (−1.252 to 1.118) | 0.316 |
|  | 18 months | −0.278 (−1.000 to 0.444) | −0.278 (−0.895 to 0.339) | 0.000 (−0.939 to 0.939) | 0.349 |
| Lawton Index | 6 months | −0.123 (−0.311 to 0.065) | 0.137 (−0.022 to 0.297) | −0.260 (−0.507 to −0.013) | 0.015 |
|  | 18 months | −0.091 (−0.266 to 0.084) | −0.019 (−0.213 to 0.176) | −0.072 (−0.331 to 0.186) | 0.220 |
| GDS-15 | 6 months | 0.333 (−0.413 to 1.079) | 0.294 (−0.409 to 0.997) | 0.039 (−0.981 to 1.059) | 0.459 |
|  | 18 months | 0.273 (−0.430 to 0.976) | −0.415 (−0.339 to 1.169) | −0.142 (−1.160 to 0.875) | 0.950 |
| Height (cm) | 6 months | −0.186 (−0.437 to 0.064) | −0.157 (−0.451 to 0.137) | −0.029 (−0.416 to 0.358) | 0.278 |
|  | 18 months | −0.377 (−0.698 to −0.056) | −0.161 (−0.409 to 0.086) | −0.216 (−0.626 to 0.195) | 0.697 |
| Body weight (kg) | 6 months | −0.277 (−0.715 to 0.160) | −0.103 (−0.834 to 0.629) | −0.175 (−0.977 to 0.627) | 0.232 |
|  | 18 months | −1.127 (−1.922 to −0.333) | 0.106 (−0.862 to 1.073) | −1.233 (−2.457 to −0.009) | 0.039 |
| Body mass index (kg/m^2^) | 6 months | −0.049 (−0.274 to 0.175) | −0.052 (−0.389 to 0.284) | 0.003 (−0.383 to 0.389) | 0.588 |
|  | 18 months | −0.222 (−0.576 to 0.133) | 0.245 (−0.269 to 0.759) | −0.466 (−1.074 to 0.141) | 0.177 |
| Fat mass (kg) | 6 months | −0.179 (−0.792 to 0.434) | 0.220 (−0.283 to 0.723) | −0.399 (−1.200 to 0.401) | 0.335 |
|  | 18 months | −0.847 (−1.582 to −0.112) | 0.131 (−0.612 to 0.872) | −0.977 (−2.001 to 0.046) | 0.055 |
| Fat-free mass (kg) | 6 months | 0.513 (−0.143 to 1.170) | 0.267 (−0.283 to 0.818) | 0.246 (−0.613 to 1.106) | 0.672 |
|  | 18 months | 0.143 (−0.735 to 1.021) | 0.025 (−0.684 to 0.734) | 0.118 (−0.987 to 1.223) | 0.733 |
| Gait speed (m/s) | 6 months | 0.002 (−0.056 to 0.060) | −0.016 (−0.070 to 0.039) | 0.018 (−0.062 to 0.097) | 0.573 |
|  | 18 months | −0.032 (−0.098 to 0.035) | −0.036 (−0.101 to 0.029) | 0.004 (−0.088 to 0.096) | 0.805 |
| One-leg standing test (s) | 6 months | 5.606 (0.940 to 10.273) | −3.316 (−8.322 to 1.691) | 8.922 (2.137 to 15.707) | 0.022 |
|  | 18 months | 3.856 (−0.914 to 8.626) | −0.373 (−6.464 to 5.718) | 4.229 (−3.260 to 11.717) | 0.161 |
| Grip strength (kg) | 6 months | −0.246 (−0.840 to 0.349) | −0.160 (−0.841 to 0.521) | −0.085 (−0.975 to 0.804) | 0.928 |
|  | 18 months | −0.102 (−0.835 to 0.631) | −0.783 (−1.511 to −0.054) | 0.681 (−0.342 to 1.704) | 0.123 |
| Fall risk index | 6 months | −0.130 (−0.744 to 0.484) | 0.000 (−0.769 to 0.769) | −0.130 (−1.093 to 0.834) | 0.383 |
|  | 18 months | 0.216 (−0.503 to 0.935) | 0.151 (−0.538 to 0.840) | 0.065 (−0.919 to 1.048) | 0.847 |
| MNA-SF | 6 months | −0.368 (−0.807 to 0.071) | −0.046 (−0.566 to 0.475) | −0.323 (−0.992 to 0.346) | 0.265 |
|  | 18 months | −0.226 (−0.830 to 0.377) | 0.302 (−0.136 to 0.740) | −0.528 (−1.265 to 0.209) | 0.177 |
| LSNS-6 | 6 months | −0.860 (−2.031 to 0.311) | 1.019 (−0.127 to 2.165) | −1.879 (−3.502 to −0.255) | 0.028 |
|  | 18 months | −1.764 (−3.094 to −0.433) | −0.537 (−1.627 to 0.553) | −1.227 (−2.930 to 0.477) | 0.601 |
| Number of participating social groups | 6 months | 0.278 (−0.087 to 0.643) | −0.220 (−0.514 to 0.074) | 0.498 (0.030 to 0.966) | 0.017 |
|  | 18 months | −0.038 (−0.443 to 0.367) | −0.208 (−0.474 to 0.059) | 0.169 (−0.308 to 0.646) | 0.116 |
| Number of medications | 6 months | −0.276 (−0.675 to 0.124) | 0.127 (−0.194 to 0.448) | −0.403 (−0.913 to 0.107) | 0.075 |
|  | 18 months | −0.035 (−0.452 to 0.381) | 0.296 (−0.110 to 0.702) | −0.331 (−0.907 to 0.245) | 0.268 |

HbA1c, glycated hemoglobin A1c; CGM, continuous glucose monitoring; CV, coefficient of variation; MAGE, mean amplitude of glycemic excursions; CONGA, continuous overall net glycemic action; MODD, mean of daily differences; TIR, time in range; TAR, time above range; TBR, time below range; GDS-15, 15-item Geriatric Depression Scale; MNA-SF, Mini-Nutritional Assessment Short-Form; LSNS-6, the Lubben Social Network Scale-6

The 95% confidence intervals for the mean changes within group and the mean differences between groups were calculated using paired and unpaired *t*-tests, respectively. The *p-*value for the mean differences between groups was calculated using the Wilcoxon rank sum test.

**Table S5. Changes in secondary categorical outcomes from baseline to 6- and 18-month follow-up in the full-analysis set**

|  | **Baseline** | | | **6-month** | | | **18-month** | | |
| --- | --- | --- | --- | --- | --- | --- | --- | --- | --- |
|  | **Intervention group** | **Control group** | ***p-*value** | **Intervention group** | **Control group** | ***p-*value** | **Intervention group** | **Control group** | **P*-*value** |
| **Self-reported hypoglycemic events** | | | | | | | | | |
| Did you experience any hypoglycemia episode that required assistance from another person in the past year?, yes | 2 (3%) | 3 (5%) | 0.578 | 1 (2%) | 2 (4%) | 0.527 | 0 (0%) | 1 (2%) | 0.298 |
| Did you experience any symptoms such as sweating, palpitation, or trembling in the past year?, yes | 5 (7%) | 3 (5%) | 0.548 | 4 (7%) | 3 (6%) | 0.751 | 4 (7%) | 3 (6%) | 0.770 |
| Did you experience any symptoms such as lightheadedness, unsteadiness, dizziness, or visual disturbance in the past year?, yes | 5 (7%) | 2 (3%) | 0.296 | 4 (7%) | 3 (5%) | 0.751 | 5 (9%) | 5 (9%) | 0.906 |
| **Microangiopathy and macroangiopathy** | | | | | | | | | |
| Retinopathy |  |  |  |  |  |  |  |  |  |
| No apparent retinopathy | 44 (70%) | 38 (70%) | 0.611 | 21 (78%) | 19 (68%) | 0.556 | 22 (76%) | 19 (68%) | 0.450 |
| Mild nonproliferative retinopathy | 13 (21%) | 9 (17%) |  | 5 (19%) | 6 (21%) |  | 5 (17%) | 4 (14%) |  |
| Moderate nonproliferative retinopathy | 5 (8%) | 7 (13%) |  | 1 (4%) | 3 (11%) |  | 2 (7%) | 5 (18%) |  |
| Severe nonproliferative retinopathy | 1 (2%) | 0 (0%) |  | 0 (0%) | 0 (0%) |  | 0 (0%) | 0 (0%) |  |
| Proliferative diabetic retinopathy | 0 (0%) | 0 (0%) |  | 0 (0%) | 0 (0%) |  | 0 (0%) | 0 (0%) |  |
| Presence of macular edema, yes | 3 (5%) | 5 (10%) | 0.292 | 1 (4%) | 4 (15%) | 0.159 | 0 (0%) | 3 (12%) | 0.074 |
| Nephropathy |  |  |  |  |  |  |  |  |  |
| Prenephropathy | 24 (41%) | 27 (52%) | 0.261 | 17 (47%) | 14 (45%) | 0.284 | 17 (47%) | 14 (41%) | 0.251 |
| Incipient nephropathy | 26 (45%) | 18 (35%) |  | 14 (39%) | 11 (35%) |  | 12 (33%) | 13 (38%) |  |
| Overt nephropathy | 8 (14%) | 5 (10%) |  | 5 (14%) | 3 (10%) |  | 7 (19%) | 4 (12%) |  |
| Kidney failure | 0 (0%) | 2 (4%) |  | 0 (0%) | 3 (10%) |  | 0 (0%) | 3 (9%) |  |
| Dialysis therapy | 0 (0%) | 0 (0%) |  | 0 (0%) | 0 (0%) |  | 0 (0%) | 0 (0%) |  |
| Achilles tendon reflex test |  |  |  |  |  |  |  |  |  |
| Normal | 29 (59%) | 28 (60%) | 0.690 | 16 (53%) | 19 (70%) | 0.343 | 15 (48%) | 22 (65%) | 0.335 |
| Decreased | 11 (22%) | 13 (28%) |  | 9 (30%) | 4 (15%) |  | 10 (32%) | 6 (18%) |  |
| Absent | 9 (18%) | 6 (13%) |  | 5 (17%) | 4 (15%) |  | 6 (19%) | 6 (18%) |  |
| Vibration test, abnormal | 18 (38%) | 20 (43%) | 0.555 | 11 (38%) | 7 (26%) | 0.337 | 15 (47%) | 13 (38%) | 0.478 |
| Presence of subjective symptoms of neuropathy, yes | 7 (14%) | 11 (24%) | 0.195 | 5 (17%) | 6 (22%) | 0.596 | 8 (24%) | 6 (18%) | 0.507 |
| **Urinary markers** | | | | | | | | | |
| Urinary glucose |  |  |  |  |  |  |  |  |  |
| Negative (-) | 48 (72%) | 37 (64%) | 0.375 | 26 (79%) | 22 (73%) | 0.209 | 25 (69%) | 21 (64%) | 0.856 |
| Trace (±) | 2 (3%) | 5 (9%) |  | 2 (6%) | 0 (0%) |  | 2 (6%) | 1 (3%) |  |
| 1+ | 3 (4%) | 1 (2%) |  | 1 (3%) | 0 (0%) |  | 1 (3%) | 1 (3%) |  |
| ≥2+ | 14 (21%) | 15 (26%) |  | 4 (12%) | 8 (27%) |  | 8 (22%) | 10 (30%) |  |
| Hematuria |  |  |  |  |  |  |  |  |  |
| Negative (-) | 47 (70%) | 40 (69%) | 0.225 | 19 (58%) | 21 (70%) | 0.623 | 25 (69%) | 26 (79%) | 0.731 |
| Trace (±) | 9 (13%) | 14 (24%) |  | 8 (24%) | 5 (17%) |  | 6 (17%) | 4 (12%) |  |
| 1+ | 9 (13%) | 3 (5%) |  | 5 (15%) | 4 (13%) |  | 2 (6%) | 2 (6%) |  |
| ≥2+ | 2 (3%) | 1 (2%) |  | 1 (3%) | 0 (0%) |  | 3 (8%) | 1 (3%) |  |
| Proteinuria |  |  |  |  |  |  |  |  |  |
| Negative (-) | 40 (59%) | 40 (69%) | 0.430 | 23 (70%) | 18 (60%) | 0.768 | 24 (67%) | 19 (58%) | 0.285 |
| Trace (±) | 15 (22%) | 9 (16%) |  | 5 (15%) | 5 (17%) |  | 10 (28%) | 7 (21%) |  |
| 1+ | 9 (13%) | 4 (7%) |  | 2 (6%) | 4 (13%) |  | 1 (3%) | 4 (12%) |  |
| ≥2+ | 4 (6%) | 5 (9%) |  | 3 (9%) | 3 (10%) |  | 1 (3%) | 3 (9%) |  |
| **Brain MR/CT imaging** | | | | | | | | | |
| Cerebral hemorrhage | 6 (9%) | 6 (10%) | 0.793 | - | - | - | 0 (0%) | 1 (3%) | 0.307 |
| Stroke | 22 (33%) | 20 (34%) | 0.846 | - | - | - | 13 (36%) | 12 (34%) | 0.872 |
| PVH (Fazekas scale) |  |  |  |  |  |  |  |  |  |
| 0 | 21 (37%) | 19 (36%) | 0.741 | - | - | - | 11 (48%) | 6 (26%) | 0.413 |
| 1 | 31 (54%) | 26 (53%) |  | - | - |  | 10 (43%) | 13 (57%) |  |
| 2 | 4 (7%) | 3 (6%) |  | - | - |  | 1 (4%) | 3 (13%) |  |
| 3 | 1 (2%) | 3 (6%) |  | - | - |  | 1 (4%) | 1 (4%) |  |
| DWMH (Fazekas scale) |  |  |  |  |  |  |  |  |  |
| 0 | 19 (34%) | 17 (36%) | 0.748 | - | - | - | 12 (52%) | 10 (43%) | 0.711 |
| 1 | 14 (25%) | 11 (23%) |  | - | - |  | 8 (35%) | 8 (35%) |  |
| 2 | 19 (34%) | 13 (28%) |  | - | - |  | 3 (13%) | 5 (22%) |  |
| 3 | 4 (7%) | 6 (13%) |  | - | - |  | 0(0%) | 0 (0%) |  |
| **Comprehensive geriatric assessment** |  |  |  |  |  |  |  |  |  |
| Physical frailty | 11 (15%) | 7 (11%) | 0.417 | 8 (11%) | 3 (5%) | 0.155 | 10 (14%) | 6 (9%) | 0.380 |
| Sarcopenia | 12 (27%) | 13 (34%) | 0.496 | 7 (21%) | 7 (25%) | 0.726 | 7 (22%) | 11 (35%) | 0.232 |

MR, magnetic resonance; CT, computed tomography; PVH, periventricular hyperintensity; DWMH, deep white matter hyperintensity

**Table S6.** **Estimated mean differences in composite score changes from baseline to the 18-month follow-up among subgroups**

|  | **Intervention group** | **Control group** | **Mean differences between groups** | **P-value** |
| --- | --- | --- | --- | --- |
| **Age at enrollment** | | | | |
| 70–77 years, *n* = 78 | 0.055 (−0.070 to 0.179) | 0.025 (−0.106 to 0.155) | 0.030 (−0.151 to 0.211) | 0.741 |
| 78–85 years, *n* = 58 | 0.023 (−0.214 to 0.261) | −0.140 (−0.402 to 0.123) | 0.163 (−0.193 to 0.519) | 0.354 |
| **Glycemic control** | | | | |
| Within the target range, *n* = 72 | −0.005 (−0.183 to 0.174) | −0.028 (−0.215 to 0.159) | 0.023 (−0.086 to 0.184) | 0.859 |
| Above the upper limit or below the lower limit, *n* = 64 | 0.086 (0.041 to 0.213) | −0.016 (−0.155 to 0.123) | 0.102 (−0.086 to 0.291) | 0.280 |
| **Drugs potentially associated with severe hypoglycemia (insulin, sulfonylurea, or glinides)** | | | | |
| Not receiving, *n* = 68 | −0.004 (−0.163 to 0.170) | −0.015 (−0.192 to 0.161) | 0.019 (−0.227 to 0.265) | 0.878 |
| Receiving, *n* = 68 | 0.085 (−0.053 to 0.223) | −0.014 (−0.161 to 0.133) | 0.099 (−0.102 to 0.300) | 0.326 |
| **APOE** | | | | |
| APOE ε4 noncarrier, *n* = 96 | 0.121 (−0.021 to 0.263) | −0.062 (−0.197 to 0.074) | 0.183 (−0.014 to 0.378) | 0.069 |
| APOE ε4 carrier, *n* = 34 | −0.031 (−0.230 to 0.168) | 0.097 (−0.176 to 0.370) | −0.128 (−0.467 to 0.210) | 0.440 |

APOE, apolipoprotein E

**Table S7. Estimated mean differences in composite cognitive score changes from baseline to 6- and 18-month follow-up based on the attendance rate of group-based physical exercise sessions (attendance rate of 50% or greater, attendance rate less than 50%, and control group)**

|  |  | **Changes within groups** | | |  | **Mean differences between groups** | | | | | |
| --- | --- | --- | --- | --- | --- | --- | --- | --- | --- | --- | --- |
|  | **Follow-up** | **Intervention group (≥50%) (*n* = 39)** | **Intervention group (<50%) (*n* = 24)** | **Control group**  **(*n* = 65)** |  | **Intervention group (≥50%) vs. Intervention group (<50%)** | ***p-*value** | **Intervention group (≥50%) vs. Control group** | ***P-*value** | **Intervention group (<50%) vs. Control group** | **P*-*value** |
| Composite score | 6 months | 0.194  (0.079 to 0.308) | -0.019  (-0.195 to 0.157) | 0.062  (-0.038 to 0.162) |  | 0.213  (-0.001 to 0.425) | 0.050 | 0.131  (-0.022 to 0.284) | 0.092 | -0.081  (-0.282 to 0.120) | 0.423 |
|  | 18 months | 0.117  (-0.008 to 0.242) | -0.095  (-0.299 to 0.120) | -0.021  (-0.133 to 0.097) |  | 0.212  (-0.030 to 0.453) | 0.085 | 0.138  (-0.037 to 0.308) | 0.107 | -0.073  (-0.305 to 0.159) | 0.531 |

**Table S8. Estimated mean differences in composite score changes from baseline to the 18-month follow-up among subgroups (post-hoc analysis)**

|  | **Intervention group** | **Control group** | **Mean differences between groups** | **P-value** |
| --- | --- | --- | --- | --- |
| **Sex** | | | | |
| Male, *n* = 88 | 0.066 (−0.087 to 0.220) | −0.004 (−0.177 to 0.170) | 0.070 (−0.162 to 0.302) | 0.547 |
| Female, *n* = 66 | 0.004 (−0.154 to 0.161) | −0.031 (−0.185 to 0.122) | 0.035 (−0.186 to 0.257) | 0.749 |
| **MMSE at enrollment** | | | | |
| MMSE ≥ 24, *n* = 127 | 0.073 (−0.029 to 0.176) | −0.023 (−0.131 to 0.085) | 0.096 (−0.054 to 0.246) | 0.205 |

MMSE, Mini-Mental State Examination

**Table S9.** **Association of changes in intakes of vitamin B1, niacin, vitamin B6, vegetables other than green-yellow vegetables, and meat with changes in the composite cognitive score and delayed recall test scores of the Rey–Osterrieth Complex Figure Test among the intervention group**

|  | **Changes in the composite cognitive score (*n* = 47)** | | |  | **Changes in the delayed recall test scores of the ROCFT (*n* = 53)** | | |
| --- | --- | --- | --- | --- | --- | --- | --- |
|  | **Coefficient (95% CI)** | ***β*** | **p-value** |  | **Coefficient (95% CI)** | ***β*** | **P-value** |
| **Nutrients** |  |  |  |  |  |  |  |
| Vitamin B1 (mg/d) | 0.501 (0.059 to 0.943) | 0.335 | 0.027 |  | 5.851 (1.189 to 10.513) | 0.326 | 0.015 |
| Niacin (mg/d) | 0.025 (−0.001 to 0.519) | 0.284 | 0.062 |  | 0.318 (0.057 to 0.579) | 0.312 | 0.018 |
| Vitamin B6 (mg/d) | 0.415 (0.112 to 0.719) | 0.394 | 0.008 |  | 3.281 (−0.084 to 6.647) | 0.258 | 0.056 |
| **Food groups** |  |  |  |  |  |  |  |
| Vegetables other than green-yellow vegetables (g/d) | 0.001 (−0.000 to 0.002) | 0.276 | 0.080 |  | 0.035 (−0.008 to 0.015) | 0.083 | 0.558 |
| Meat (g/d) | 0.002 (−0.002 to 0.005) | 0.166 | 0.322 |  | 0.031 (−0.002 to 0.064) | 0.260 | 0.062 |

Multiple regression analyses adjusted for baseline age, sex, years of education, composite cognitive score, delayed recall test scores of the ROCFT were performed.

ROCFT, Rey–Osterrieth Complex Figure Test

**Table S10. Serious adverse events developed during the trial**

|  | **Overall (*n* = 136)** | **Intervention group (*n* = 71)** | **Control group (*n* = 65)** |
| --- | --- | --- | --- |
| All severe adverse events | 36 | 28 | 8 |
| At least one serious adverse event | 30 (22%) | 22 (31%) | 8 (12%) |
| Gastrointestinal disorders | 1 (1%) | 1 (1%) | 0 (0%) |
| Cardiac disorders | 2 (2%) | 2 (3%) | 0 (0%) |
| Eye disorders | 10 (7%) | 8 (11%) | 2 (3%) |
| Musculoskeletal and connective tissue disorders | 1 (1%) | 1 (1%) | 0 (0%) |
| Infections and infestations | 3 (2%) | 1 (1%) | 2 (3%) |
| Metabolism and nutrition disorders | 5 (4%) | 3 (4%) | 2 (3%) |
| Injury, poisoning, and procedural complications | 7 (5%) | 6 (8%) | 1 (2%) |
| Blood and lymphatic system disorders | 2 (2%) | 2 (3%) | 0 (0%) |
| Neoplasms (benign, malignant, and unspecified) | 2 (2%) | 2 (3%) | 0 (0%) |
| Skin and subcutaneous tissue disorders | 1 (1%) | 1 (1%) | 0 (0%) |
| Nervous system disorders | 2 (2%) | 1 (1%) | 1 (2%) |


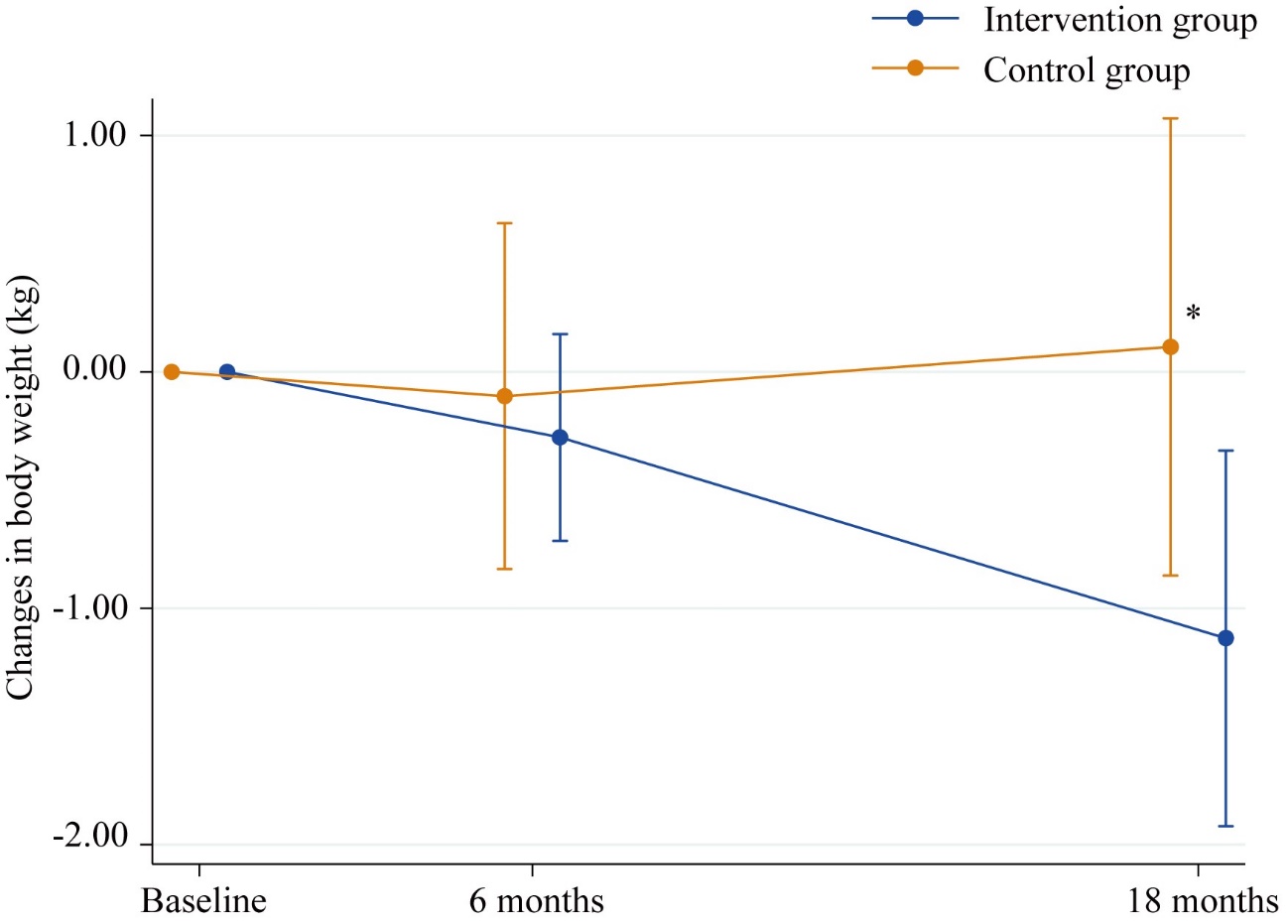


**Figure S1. Changes in body weight from baseline to 18-month follow-up**

*The mean difference in body weight changes between the intervention and control groups was statistically significant (P < 0.05).


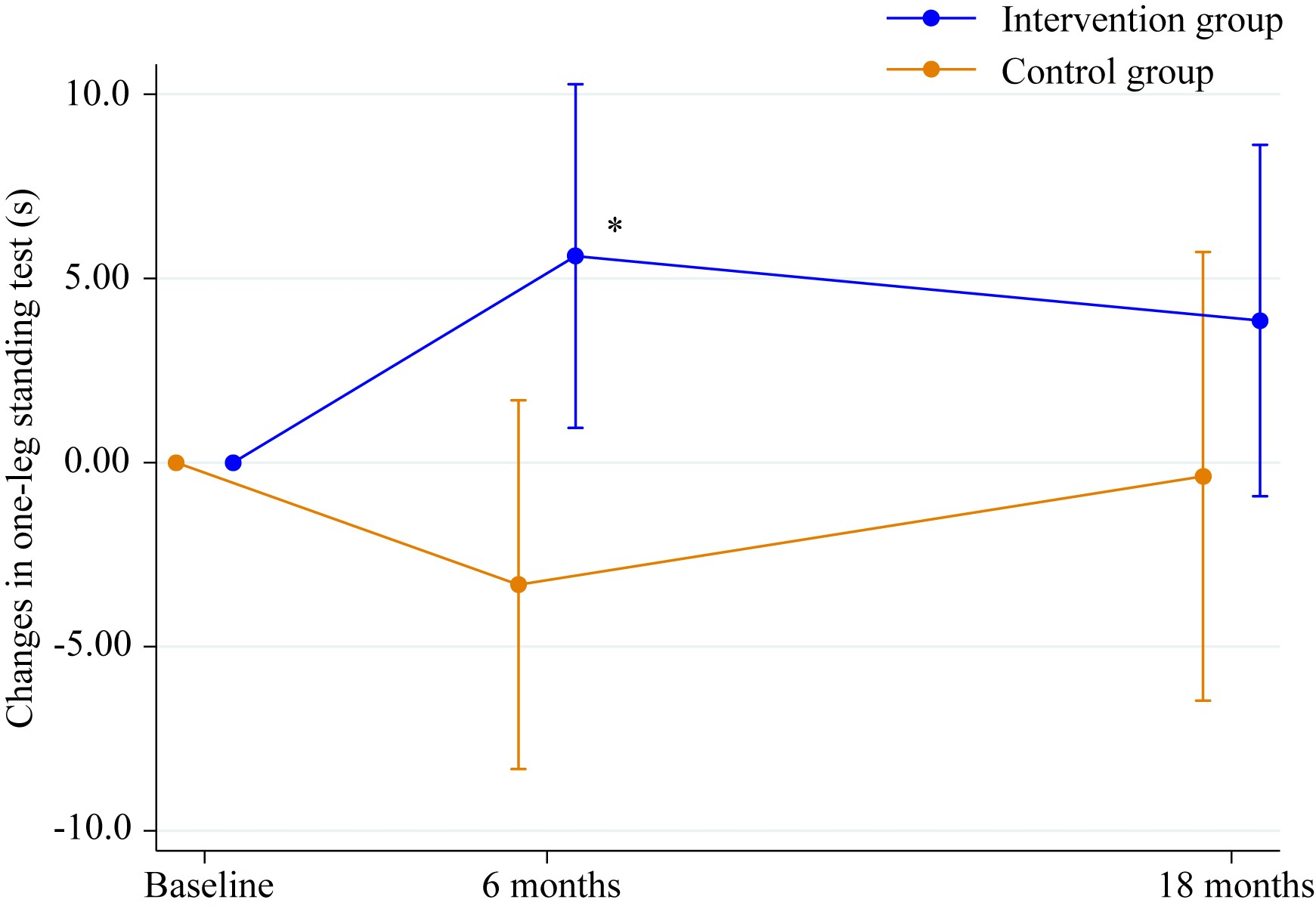


**Figure S2. Changes in the performance of the one-leg standing test from baseline to 18-month follow-up**

*The mean difference in changes in the one-leg standing test between the intervention and control groups was statistically significant (P < 0.05).


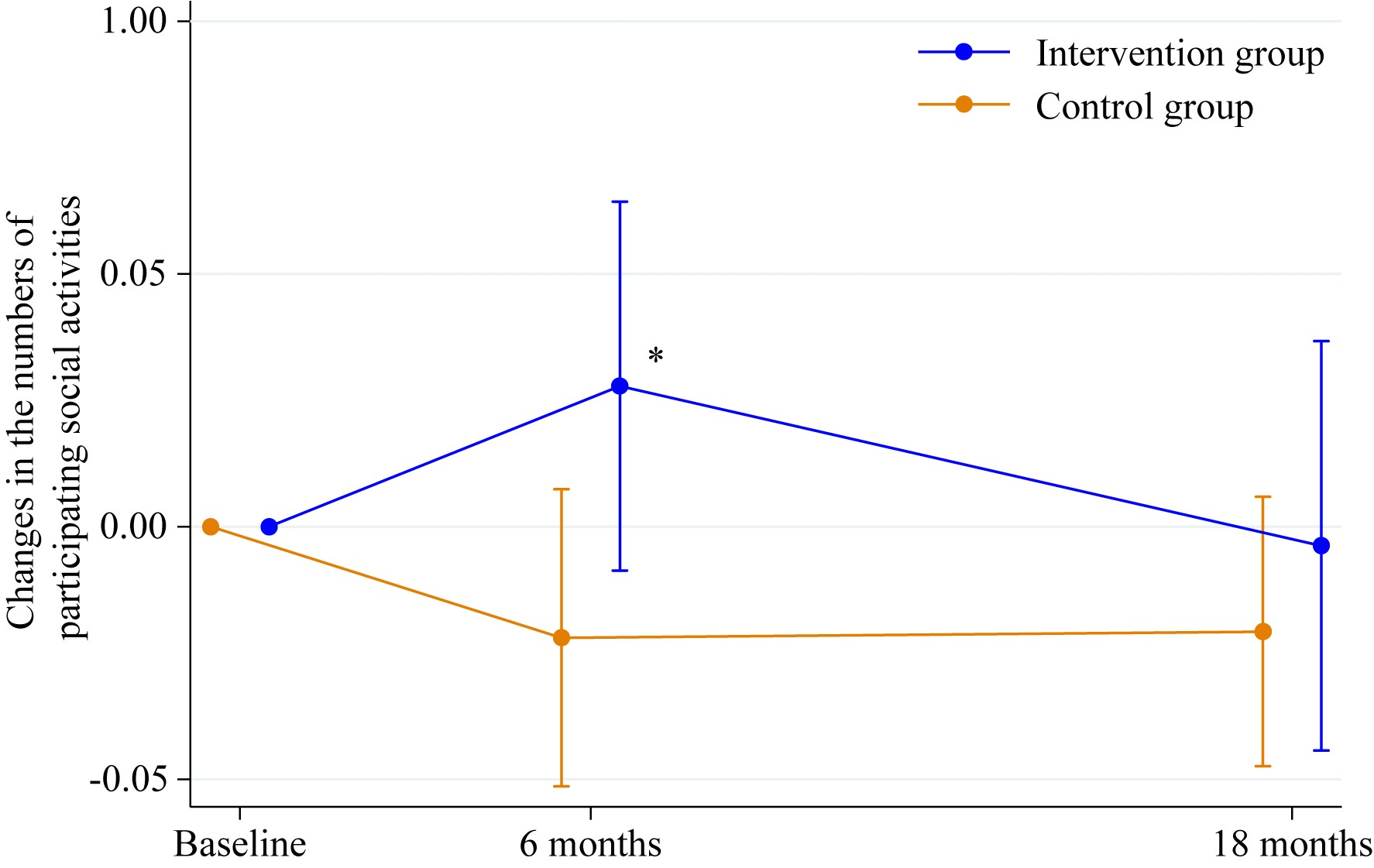


**Figure S3. Changes in the number of participating social groups from baseline to 18-month follow-up**

*The mean difference in changes in the number of participating social groups between the intervention and control groups was statistically significant (P < 0.05).


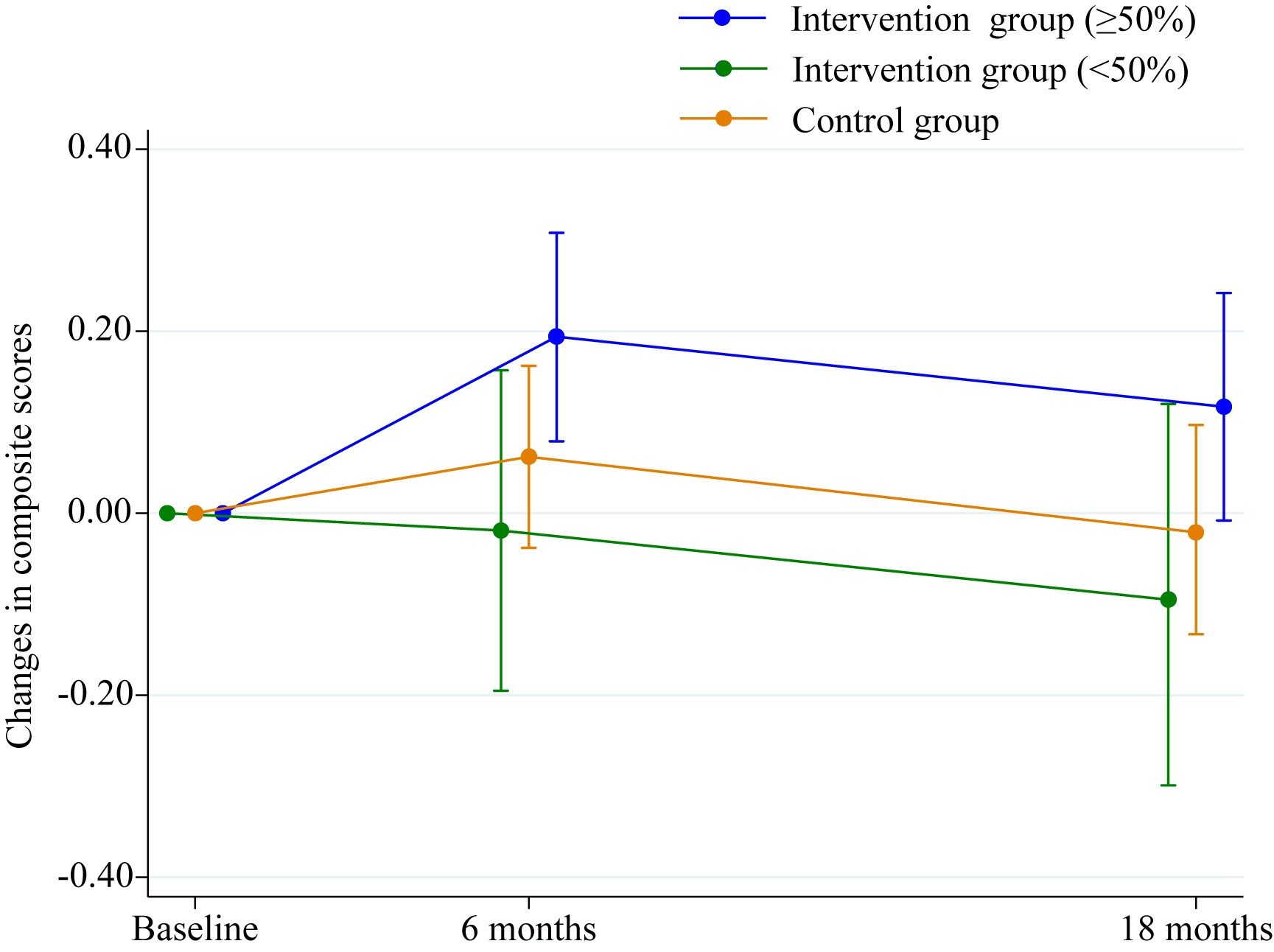


**Figure S4. Changes in composite scores from baseline to 18-month follow-up** **based on the attendance rate of group-based physical exercise sessions (attendance rate of 50% or greater, attendance rate less than 50%, and control group)**
